# Observation of Localized Resonant Phonon Polaritons in Biaxial α‐MoO_3_ Nanoparticles

**DOI:** 10.1002/advs.202417123

**Published:** 2025-09-15

**Authors:** Daniel Beitner, Asaf Farhi, Ravindra Kumar Nitharwal, Tejendra Dixit, Tzvia Beitner, Shachar Richter, SivaRama Krishnan, Haim Suchowski

**Affiliations:** ^1^ Department of Materials Science and Engineering Faculty of Engineering Tel Aviv University Tel Aviv 6997801 Israel; ^2^ University Centre for Nanoscience and Nanotechnology Tel Aviv University Tel Aviv 6997801 Israel; ^3^ School of Physics and Astronomy Faculty of Exact Sciences Tel Aviv University Tel Aviv 6997801 Israel; ^4^ Department of Physics and Quantum Center of Excellence for Diamond and Emerging Materials Indian Institute of Technology Madras Chennai 600036 India; ^5^ Department of Electronics and Communication Engineering Indian Institute of Information Technology Design and Manufacturing (IIITDM) Kancheepuram 600127 India

**Keywords:** anisotropic materials, light‐emitting materials, liquid crystals, magnetic materials, materials science, nanomaterials, nanoparticles, nanotechnology, optics, photonics, polaritons, sensors

## Abstract

Anisotropic subwavelength particles uniquely combine the strong, tunable response of nanostructures with the exotic properties of anisotropic materials, enabling diverse applications in photonics, biomedicine, and magnetism. Anisotropic particles are also prevalent in systems such as ice grains, liquid crystal droplets, and ferromagnetic particles. Nanostructures supporting hyperbolic phonon‐polaritons hold significant promise for infrared applications due to their strong anisotropic optical response. However, previous experiments primarily explored isotropic or uniaxial nanostructures, with eigenmode theories limited to isotropic particles, restricting the understanding and applicability of anisotropic particles. Here, localized phonon resonances in the mid‐infrared spectral region in biaxial nanoparticles with three distinct axial permittivities are observed. Using a novel femtosecond‐pulsed laser ablation method, α‐molybdenum trioxide nanoparticles are synthesized with tunable, high‐Q‐factor mid‐infrared resonances. Additionally, a comprehensive theoretical framework is derived for anisotropic nanoparticles, which aligns exceptionally well with the experimental results. The findings uncover the physics of polaritons in biaxial nanoparticles, including both fundamental and higher‐order modes, paralleling the significant shift in isotropic plasmon‐polariton research toward nanostructure resonators in the visible range. The research paves the way for a new generation of tunable, multispectral, anisotropic, and directional mid‐infrared nanoresonators, opening new possibilities for mid‐infrared imaging, sensitive photonic devices, and biomarkers.

## Graphical abstract

Biaxial α‐MoO_3_ nanoparticles exhibit tunable, high‐Q mid‐infrared resonances driven by localized phonon polaritons. Combining a novel synthesis method, hyperspectral near‐field imaging and a unified theoretical analysis, this work reveals eigenmodes and resonance conditions in biaxial particles. These findings unlock the potential for a new generation of directional, multispectral mid‐IR nanophotonics, with broad implications for imaging, sensing, and magnetostatics.

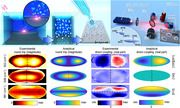

## 1 Introduction

When optical phonons, that is, out‐of‐phase lattice vibrations in polar crystalline materials, are coupled to photons, they give rise to quasiparticles known as phonon polaritons (PhPs). These PhPs have been extensively studied in polar dielectrics,^[^ ^1^ ^]^ polar semiconductors,^[^ ^1^ ^]^ and recently in 2D materials.^[^ ^2^, ^3^ ^]^ The potential of PhPs to manipulat light at the nanoscale, especially in the mid‐infrared (mid‐IR) spectral range, is critical for the development of novel IR‐active metamaterials technologies.

Hyperbolic PhPs (HPhPs) arise in highly anisotropic materials when the real parts of the permittivity have opposite signs along different directions.^[^ ^4^, ^5^ ^]^ They have recently garnered considerable attention due to their tightly confined and directional nature. These characteristics make HPhPs highly advantageous for various optical applications, including enhanced spontaneous emission^,[^ ^6^, ^7^ ^]^ hyper‐lensing,^[^ ^8^, ^9^, ^10^, ^11^, ^12^, ^13^ ^]^ high‐sensitivity chemical sensing,^[^ ^14^, ^15^ ^]^ negative refraction,^[^ ^16^, ^17^, ^18^ ^]^ and waveguiding.^[^ ^19^, ^20^ ^]^ Such anisotropic materials exhibit unique mechanical, electrical, thermal, and optical behaviors. In addition, their directional and multispectral nature unlocks potential applications across these fields.^[^ ^21^, ^22^ ^]^

Recently, several naturally anisotropic polar materials that support HPhPs, such as hexagonal boron nitride (h‐BN),^[^ ^3^, ^23^, ^24^, ^25^ ^]^ α‐phase molybdenum trioxide (α‐MoO_3_),^[^ ^26^ ^]^ and α‐phase vanadium pentoxide,^[^ ^27^ ^]^ have been identified. Among these, α‐MoO_3_ has gained notable interest due to its biaxial anisotropy characterized by distinct optical indices along each axis and long‐lived HPhPs active in the atmospheric mid‐IR window. Diverse strategies for modifying HPhPs in α‐MoO_3_ have been explored, including adjustments to layer thickness,^[^ ^26^, ^28^ ^]^ inter‐layer twist angle,^[^ ^29^, ^30^, ^31^ ^]^ coupling with plasmonic nanoparticles,^[^ ^32^, ^33^ ^]^ and geometric confinement.^[^ ^5^, ^26^, ^34^, ^35^, ^36^, ^37^, ^38^ ^]^

When materials are shaped into nanostructures, their optical response can be tuned by adjusting their geometry^[^ ^39^ ^]^ and their response can be strong, since their resonance condition does not require gain.^[^ ^40^ ^]^ While isotropic nanostructures have been widely used in various applications, such as detectors and biomarkers, anisotropic‐material nanostructures are at the forefront of photonics research, holding promise for a new generation of anisotropic resonators. To date, HPhPs in α‐MoO_3_ have been demonstrated in flakes,^[^ ^29^, ^31^, ^33^, ^37^, ^41^ ^]^ macro‐disks,^[^ ^26^, ^35^ ^]^ nanobelts,^[^ ^5^, ^38^, ^42^ ^]^ nanocavities,^[^ ^32^ ^]^ and metamaterials.^[^ ^36^, ^43^ ^]^ While recent works on subwavelength geometries have demonstrated the existence of localized phonon‐polariton (LPhP) resonances in uniaxial materials, such as hBN for nanodisks,^[^ ^39^ ^]^ nanobars,^[^ ^44^ ^]^ and nanopillars,^[^ ^25^ ^]^ research on phonon‐polaritons in biaxial materials has centered solely on *propagating* HPhP modes on 2D surfaces or large macro‐sized particles. The optical response and applications of biaxial localized nanostructures have remained entirely unexplored.

Here, we introduce a new class of mid‐IR biaxial nanoparticle resonators exhibiting extremely high‐quality factors with controlled directionality. We present the first experimental observation of LPhPs in subwavelength biaxial nanoparticles composed of a hyperbolic material. As part of our research, we have utilized a novel method of femtosecond‐pulsed laser ablation in a liquid (fs‐PLAL) to synthesize α‐MoO_3_ nanoparticles. This is the first reported example of synthesized nanoellipsoid biaxial particles with mid‐IR resonances. We have fully characterized these biaxial nanoparticles spatially and spectrally using a mid‐IR scattering‐scanning near‐field optical microscope (s‐SNOM) and showed that our α‐MoO_3_ nanoparticles have various geometries and a diverse optical spectrum of phononic polaritons spanning the 900‐1000 cm^−1^ range. The varied geometries of these nanoparticles allow for broad tunability of the resonances of the LPhPs, including multiple modes and high‐order directional polaritons.

To explain our observations, we developed a novel theory of general anisotropic subwavelength particle modes and eigen‐permittivity relations. This theory predicts resonance sum rules for the axial permittivities, offering insights into the response and design of anisotropic particles. We observe the predicted LPhP modes, oriented both along individual principal crystal axes and with respect to two axes, with coupling between the permittivities of different crystal axes. These nanoparticles could usher in a new era of detectors, image sensors, and highly sensitive photonic devices in the mid‐IR range.

## 2 Synthesis of α‐MoO_3_ Nanoparticles

**Figure 1:**
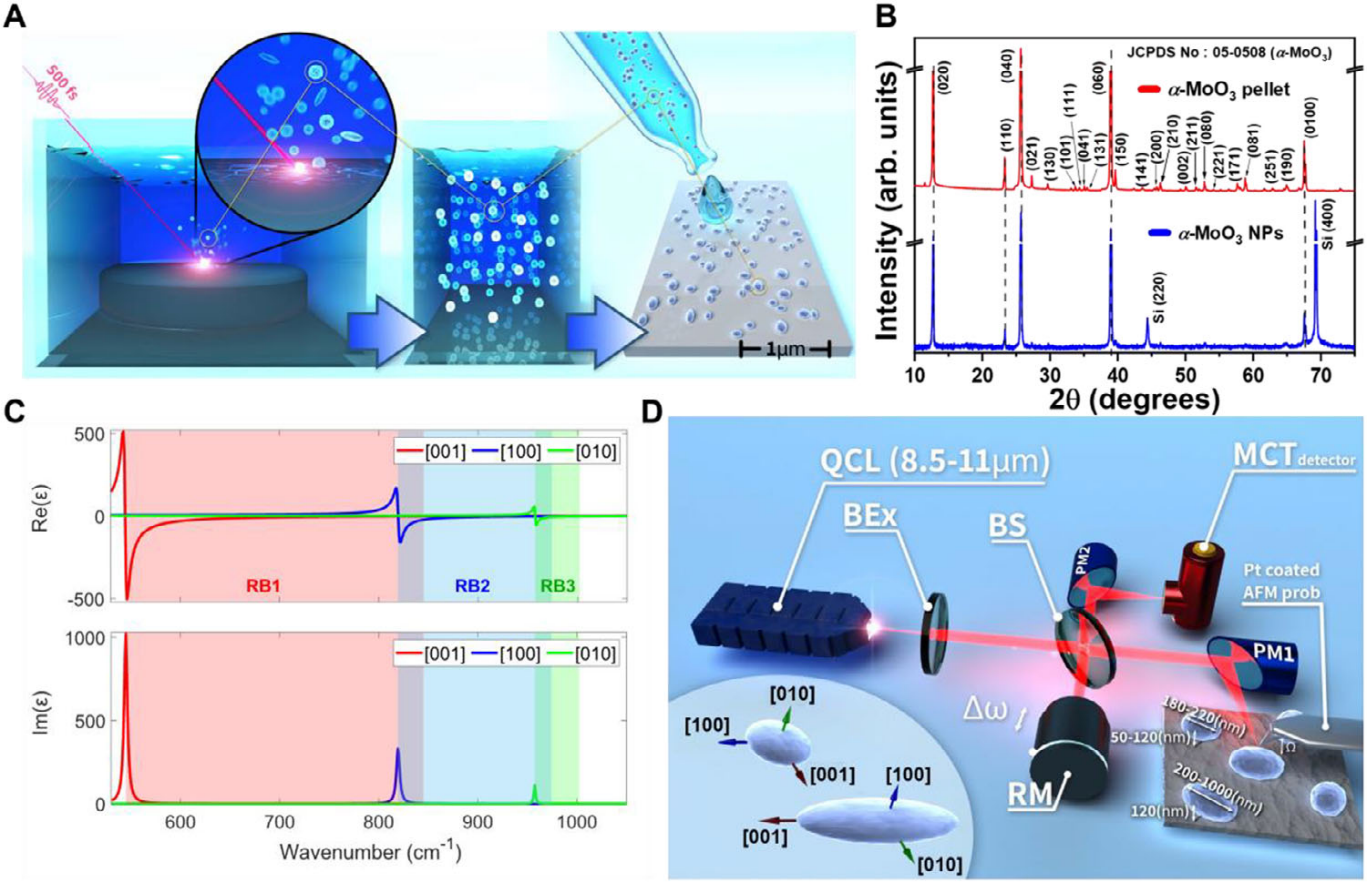
fs‐PLAL synthesis process of creating α‐MoO nanoparticles and their characterization. A) – Schematic of the fs‐PLAL process: In the first stage, the fs laser is focused on the surface of a slab of α‐MoO_3_ and ablates the nanoparticles. The nanoparticles are washed in deionized water and drop‐cast onto a Si surface. B) X‐ray diffraction pattern of α‐MoO_3_ nanoparticles (black line) and α‐MoO_3_ pellet (red line), showing an excellent match to the α‐MoO_3_ phase. C) Calculated real and imaginary parts of the dielectric permittivity of α‐MoO_3_ along the three crystal directions ([010], [001], and [100]).^[^ ^45^ ^]^ The shaded regions indicate the areas of the three Reststrahlen bands. D) Simplified s‐SNOM schematic with the components: quantum cascade laser (QCL), beam expander (BEx), beam splitter (BS), parabolic mirror 1 and 2 (PM), reference mirror (RM) oscillating at ***Δω***, Pt‐coated AFM probe oscillating at ***Ω***, and HgCdTe (MCT) liquid N_2_‐cooled amplified photodetector. The two studied α‐MoO_3_ nanoparticles, along with the crystal directions presumed from measurements, are presented.

α‐MoO_3_ nanoparticles were synthesized using fs‐PLAL, which enables the production of particles of varying sizes. **Figure** **1**A shows a schematic of the process. A slab of the material is placed in acetone, and an fs laser source is focused on its surface. The laser pulses ablate the material to create nanoparticles. After ablation, the particles are dispersed in deionized water, cleaned, and deposited onto a Si substrate through drop casting. X‐ray diffraction and Raman spectra from the ablated particles confirm their composition as α‐MoO_3_, as shown in Figure 1B and in the supplementary information (SI), Figures S1–S3 (Supporting Information).

α‐MoO_3_ is a biaxial hyperbolic material with three distinct Reststrahlen bands (RB) in each crystal direction of its unit cell. Within the spectral range of the RB band, the real dielectric value becomes negative in that crystal direction. Specifically, the lowest energy band, RB1 (545‐851 cm^−1^), extends along the [001] direction, RB2 (822–962 cm^−1^) extends in the [100] direction, and RB3 (957–1007 cm^−1^) is aligned with the [010] direction, as seen in Figure 1C. The ablated α‐MoO_3_ particles tend to approximately take the form of either small oblate nanoellipsoids with a minor axis diameter of 50‐120 nm and two equal large axes diameters of 180‐220 nm, or prolate ellipsoids with a thickness of ≈120 nm and lengths ranging from 200 to 1000 nm, as seen in the inset in Figure 1D. The dimensions of the nanoellipsoids were measured using both atomic force microscope (AFM) topological measurements and scanning electron microscopy imaging, as seen in Figures S3 and S6 (Supporting Information). The directions of the crystal axes of the two nanoellipsoids were determined by comparing the measured near‐field modes and resonance shapes to the analytical theory (see Figures S4 and S5, Supporting Information). Specifically, we determined the crystal orientations with a high degree of certainty by examining the measured spatial near‐field data at the resonance wavelengths of the [100] and [010] dipole modes.

## 3 Hyperspectral Near‐Field Characterization of the Nanoparticles

The s‐SNOM measurement system used in this study is illustrated in Figure 1D. A tunable continuous‐wave quantum cascade laser is used to illuminate a metallic‐coated AFM probe in tapping mode. The light scattered from the AFM probe tip is collected in a pseudo‐heterodyne detection scheme. A lock‐in amplifier separates the near‐field signal from the far‐field noise by demodulating the total signal to higher harmonics of the probe tapping frequency. Specifically, the near‐field optical data presented in this work were taken from the third harmonic of the signal. The pseudo‐heterodyne detection scheme allows for recording the complex optical signal, denoted by $\sigma_{n}=S_{n}\cdot e^{\phi_{n}i}$, where σ_*n*_, *S_n_* and ϕ_*n*_ represent the *n* ^th^ order harmonic of the s‐SNOM total signal, amplitude, and phase, respectively.^[^ ^46^ ^]^ We used the s‐SNOM to image both the sample topological features and to spatially image the different resonance modes, recording the optical phase and amplitude spectra in the 850‐1050 cm^−1^ (8.5‐11 µm) spectral range. Due to the use of lock‐in amplification, only optical signals modulated by the AFM probe tip are measured.

**Figure 2:**
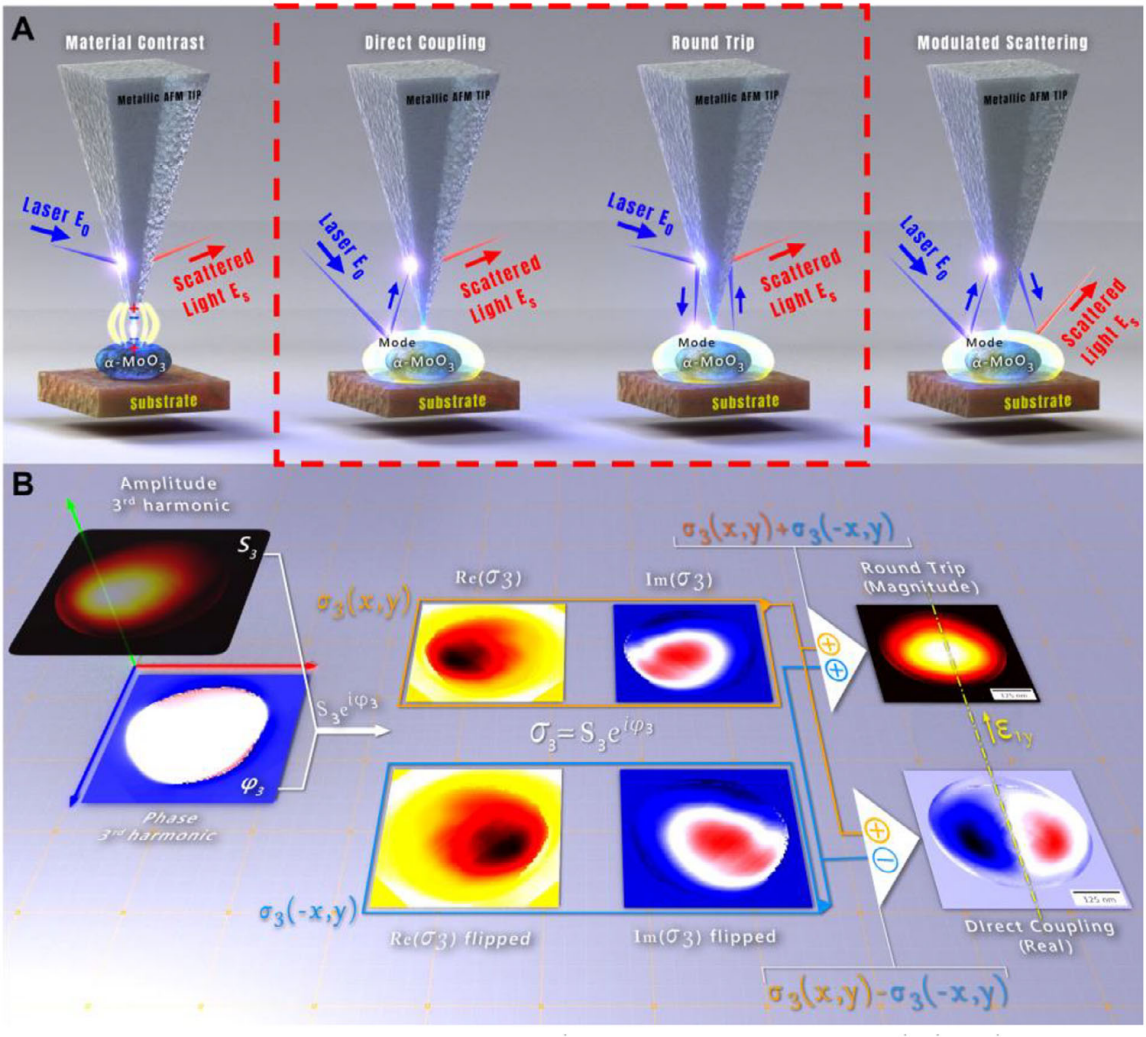
Different components of the measured s‐SNOM image and the decomposition method used in the study to separate round‐trip and direct coupling components. A) Schematic showing the interaction paths of the different components of the measured s‐SNOM image. The measured complex s‐SNOM image is a superposition of these components. The material contrast component arises from the interaction between the dielectric properties of the sample, independently of its geometry, and the dipole induced by the AFM tip. The direct coupling component arises from the excitation of the resonant mode by the far field and its measurement by the AFM tip. It has a single AFM tip‐mode interaction (small blue arrows) and the measured signal scales as E_z_(r_0_), typically resulting in an antisymmetric image for in‐plane modes. The round‐trip component is due to excitation and measurement of the resonant mode via the AFM tip. It has two AFM tip‐mode interactions (small blue arrows), causing it to scale as E_z_(r_0_)^2^ and resulting in a symmetrical image. The modulated scattering component is due to the particle modes excited and measured via the far field but modulated via interaction with the AFM tip. For single resonant nanoparticles, the modulated scattering component is negligible. The two paths marked by the dashed red line are the main contributions to the mode image (B). The method used to decompose the different components of the complex s‐SNOM images. Initially, the measured s‐SNOM amplitude (S_3_) and phase (φ_3_) images, demodulated to the 3_rd_ harmonic of the AFM probe resonance, are combined to obtain the complex s‐SNOM image σ_3_(x,y). σ_3_(x,y) is then separated into its real Re(σ_3_) and imaginary Im(σ_3_) components, and a copy of the image is created and flipped along the appropriate crystal axis for the extracted mode σ_3_(‐x,y). The two image sets are then summed to extract the symmetrical component (round‐trip) or subtracted to extract the antisymmetric component (direct coupling). For modes along the AFM probe's z direction (perpendicular to the scanning plane), the contribution of direct coupling will vanish by definition.

The complex near‐field signal resulting from the interaction between the probing tip and the resonant structure, such as the α‐MoO_3_ nanoparticles studied in this work, can be decomposed into four main components, as seen in **Figure** **2**A.^[^ ^39^ ^]^ The material contrast component arises from the dipole induced by the AFM tip onto the material below. This is the contribution of the non‐resonant local dielectric environment near the AFM tip, and represents the polarizability of the material as an infinite layer. Its value across the particle surface is uniform, as it only depends on the material properties directly under the AFM tip. Thus, its effect on the s‐SNOM image is a uniform contrast difference between α‐MoO_3_ and the substrate, which is subtracted from the resonant mode image to improve the contrast between the mode and the background. The direct coupling (DCo) component originates from the modes excited by the incident beam and then scattered to the far field through coupling with the AFM tip. The magnitude of the DCo image depends on the polarization direction of the incident beam relative to the direction of the excited mode. The signal measured by the tip scales as the *z* component of the electric field of the mode at the tip location *E_z_* (**r**_0_), which is usually an odd function with respect to a crystal axis, as explained in the Supporting Information.^[^ ^36^, ^41^ ^]^

The round‐trip (RTr) component involves the modes that are excited and subsequently scattered by the AFM tip. In this case, the mode couples twice to the tip—once during excitation and once during measurement, as seen in Figure 2A. As a result, the measured signal scales as *E_z_* (**r**_0_)^2^_,_ which is an even function with respect to a crystal axis (see Supporting Information for details). In one case, that of the out‐of‐plane dipole, *E_z_* (**r**_0_) is even, and hence both the near and far fields are symmetric; see Supporting Information for details.

The modulated scattering involves the excitation of the resonant mode in the nanoparticle, which emits directly to the far field but is perturbed by the AFM tip, resulting in a modulated signal that can be measured by the s‐SNOM. Experimentally, this contribution was found to be negligible for single particles.^[^ ^39^ ^]^ While the tip‐substrate coupling can affect the SNOM measurement, in our case, the tip is distanced more than 50 nm from the substrate; therefore, its effect is expected to be negligible.^[^ ^47^ ^]^

For ellipsoid‐shaped nanoparticles, where the crystal axes align with the geometrical axis, the RTr contribution will exhibit an even symmetry with respect to the eigenmode direction. In DCo, the in‐plane modes excited via the AFM tip have odd symmetry. The complex s‐SNOM signal can thus be decomposed into these DCo and RTr contributions using their symmetry properties (see details in the Methods Section and Ref.[39] which considers isotropic modes). We can calculate DCo (RTr) by subtracting (adding) the replica mirrored across the symmetry axis from (to) the complex near‐field image, as seen in Figure 2B. Notably, unlike isotropic modes,^[^ ^36^, ^39^ ^]^ our modes are directional, and we have adjusted the analysis accordingly. Specifically, our symmetry axes are the resonant crystal axes along which the modes are excited, as opposed to the field polarization axis, as in Ref.[39] The Q‐factor of the resonance for the modes was calculated using the frequency‐to‐bandwidth ratio in the resonance peak of the near‐field phase, summed over the area of the particle.

## 4 Analytical Eigenmode Theory of Anisotropic Nanoparticles

To calculate the eigenstates of anisotropic particles in the quasistatic regime, we solve Laplace's equation^[^ ^40^, ^48^ ^]^ for an anisotropic medium without a source. The equation is $\;\nabla \cdot \overset{↔}{\varepsilon }\;\nabla \psi_{n}=0$, where ψ_*n*_ is an electric potential eigenstate and $\overset{↔}{\varepsilon }$ is the eigen‐permittivity tensor. The permittivity tensor equals ε_1_ inside the inclusion, representing the material's intrinsic dielectric properties, and ε_2_ = *I* in the host medium, where *I* is the identity matrix, indicating a homogeneous and isotropic environment.^[^ ^49^ ^]^

We first expressed the dipole‐mode eigenstates and eigen‐permittivities for an anisotropic sphere and ellipsoid based on the scattering analyses described in the literature^[^ ^50^, ^51^ ^]^

(1) $${\tilde{\psi }}_{\mathrm{sphere},\;\;l=1,m=0}=\frac{1}{\sqrt{a}}\left\{\begin{array}{cc} \frac{r}{a}\cos\theta & r<a\\ \frac{a^{2}}{r^{2}}\cos\theta & r\geq a \end{array},\in_{1z}=-2\right.$$

(2) $${\tilde{\psi }}_{l=1,m=0,\mathrm{inside}\;\mathrm{ellipsoid}}\propto z,\in_{1z}=\in_{2}\left(L_{z}-1\right)/L_{z}$$

(3) $$L_{z}=\frac{abc}{2}\int_{0}^{\infty }\frac{dq}{\left(c^{2}+q\right)R\left(q\right)},R\left(q\right)\equiv {\left[\left(q+a^{2}\right)\left(q+b^{2}\right)\left(q+c^{2}\right)\right]}^{1/2}$$

where, in the case of an ellipsoid, *a*, *b*, *c* are its intersections with the positive *x*, *y*, *z* axes, respectively. The first‐order eigenstates and eigen‐permittivities are identical to those of isotropic modes. However, they are directional and can only be oriented along the resonant crystal directions of the α‐MoO_3_ crystal, regardless of the field polarization direction, as long as it has a polarization component along the relevant axis.

We qualitatively analyzed these dipole resonances for an anisotropic ellipsoid inclusion with crystal directions aligned along its geometrical axes, to determine the impact of its axis length aspect ratios on the resonance behavior.^[^ ^45^ ^]^ We observed from the expressions for the resonant permittivities above that an elongated (shortened) axis results in a lower (higher) eigen‐permittivity value compared to a sphere. Since the real part of the material's permittivity increases monotonically with frequency in the resonant negative permittivity range, the permittivity will approach the axial eigen‐permittivity at lower (higher) frequencies, resulting in redshifted (blueshifted) resonances. Material permittivity values with small real parts have large imaginary parts (see Figure 1C), which weaken the resonance, since the eigen‐permittivity is generally real.^[^ ^40^ ^]^ Thus, a shortened ellipsoid axis, which is associated with a higher axial eigen‐permittivity and blueshifted resonance, results in a more pronounced resonance peak.

Next, we analyzed the higher‐order anisotropic sphere modes and eigen‐permittivity relations. These higher‐order modes are important because they play a dominant role in interactions at distances shorter than the wavelength, such as near‐field excitation or when multiple particles in close proximity are excited via a far field, affecting the overall resonance behavior. We expressed the eigenstates in Cartesian coordinates due to the crystal's symmetry. These eigenstates satisfy both the boundary conditions and Laplace's equation, applicable to anisotropic media (i.e., those where $\in_{i}\neq \in_{j}$). The field outside the sphere can be expanded into spherical harmonics. However, the anisotropic nature of the material prevents the straightforward use of high‐order spherical harmonics inside the particle. Since there is continuity of the potential on the particle envelope, the potential on the particle interface can be expanded with spherical harmonics, which is highly restrictive for the choice of the eigenstates inside the particle. Therefore, we suggest that the anisotropic modes are specific superpositions of isotropic‐sphere modes. For biaxial particles one can choose superpositions of modes for which $\frac{\partial^{2}\tilde{\psi }}{\partial x_{i}^{2}}=\frac{\partial^{2}\tilde{\psi }}{\partial x_{j}^{2}}=0.$ This is because for this choice the two axial permittivity components ε_1*i*_ and ε_1*j*_ do not contribute to Laplace's equation, and it can be treated as an isotropic Laplace's equation. Clearly, these modes also satisfy Laplace's equation in the isotropic medium outside the particle.

We thus find the following second‐order anisotropic modes for biaxial spheres, along with eigen‐permittivity relations:

(4) $${\tilde{\psi }}_{2}^{2}=\frac{\left(\psi_{2}^{1}-\psi_{2}^{-1}\right)}{2}\overset{\mathrm{inside}}{\to }xz$$

(5)

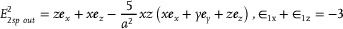

where ψ denotes isotropic sphere modes, $\tilde{\psi }$ denotes anisotropic sphere modes, and the first transitions are for inside the sphere volume. Here, the second‐order anisotropic ellipsoid mode, is associated with two axes, as can be seen in ${\tilde{\psi }}_{2}^{2}$, unlike the dipole mode. Interestingly, there is coupling between the permittivities of the different axes via sum rules for the second‐order modes, unlike in the discrete eigen‐permittivities for isotropic particles.^[^ ^48^, ^49^ ^]^ In addition, as opposed to particles with geometric or material isotropy,^[^ ^52^ ^]^ the modes are not degenerate. As a result, they are directional and are not affected by the source, except for their excitation magnitude.

Similarly, for the ellipsoid particle, we find the same modes inside the inclusion volume and the following mode field outside the ellipsoid and the eigen‐permittivity sum rule:

(6)

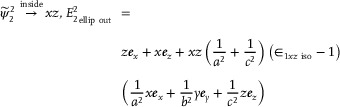

(7) $$\frac{c^{2}\in_{1x}+a^{2}\in_{1z}}{a^{2}+c^{2}}=\in_{1xz\;\mathrm{iso}},\;\in_{1xz\;\mathrm{iso}}=1-{\left(\frac{a_{1}a_{2}a_{3}}{2}\left(a_{x}^{2}+a_{z}^{2}\right)I_{xz}\right)}^{-1}$$

where $I_{\alpha \beta }=\int_{0}^{\infty }\frac{du}{\left(u+a_{\alpha }^{2}\right)\left(u+a_{\beta }^{2}\right)R\left(u\right)}$, and in Ref.[50] the eigen‐permittivity was calculated via $\in_{1xz\;\mathrm{iso}}=1+\frac{1}{4\pi \psi_{n}\left(r\right)}\int_{V}dr^{\prime }\nabla^{\prime }\psi_{n}\left(r^{\prime }\right)\nabla \left(\frac{1}{r-r^{\prime }}\right)$ (*V* is the ellipsoid volume). Note that while we assumed uniform axial permittivities, nanoparticles have large surface‐to‐volume ratios. Since surface effects can modify the local dielectric response near the surface, they may contribute to a slight shift in the observed resonances, which could explain a small discrepancy between the experimental and theoretical results. To incorporate this effect, one may utilize effective medium theory or consider a core‐shell ellipsoidal structure, where the shell represents a surface layer of the ellipsoid with a different effective permittivity.^[^ ^53^, ^54^ ^]^

**Figure 3:**
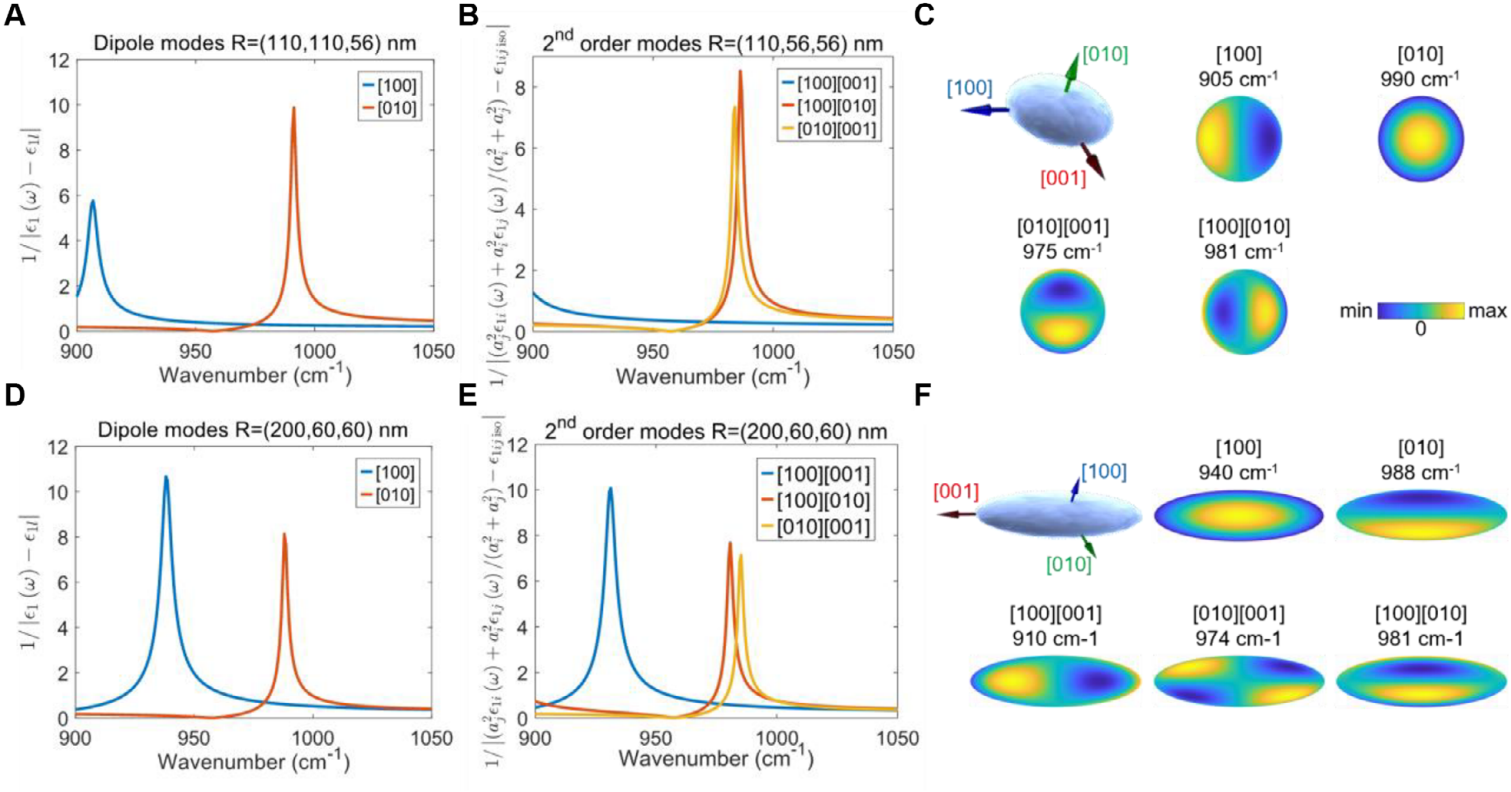
Spectra and electric field mode projection on the s‐SNOM tip from the theoretical analysis for the two α‐MoO_3_ nano‐ellipsoids. A,B) First‐ and second‐order mode scattering of the R=(110110,56) nm oblate nano‐ellipsoids with the AFM probe aligned along the α‐MoO_3_ *z*‐[010] with the crystal configuration shown in Figure 1D, upper image. C) Two‐dimensional (in the *x*‐[100] *y*‐[001] plane) projections of the real part of the electric field mode on the s‐SNOM tip. D,E) First‐ and second‐order mode scattering for the prolate R=(200,60,60) nm nanoellipsoids with the AFM probe aligned along the α‐MoO_3_ *z*‐[100] with the crystal configuration shown in Figure 1D, lower image. F) Two‐dimensional (in the *x*‐[001] *y*‐[010] plane) projections of the modes of the second ellipsoid on the s‐SNOM tip.

**Figure** **3**A,B,D, and E show the predicted scattering peaks for the first‐ and second‐order modes of oblate ellipsoids with *R* = (110110,56) nm and *R* = (200,60,60) nm. Figure 3C,F shows the predicted projections of these modes on the s‐SNOM probe tip *E_z_* (*r*_0_) when it is aligned along the α‐MoO_3_ *z* direction; see SI p. 14‐17. The resulting mode spectra for the other crystal orientations were also calculated (see Figures S4 and S5, Supporting Information). Finally, we also calculated the near‐field contribution of the modes via $E_{z}^{2}\left(r_{0}\right)$, as explained above. In addition to the 1^st^ and 2^nd^ order mode results, our theoretical framework can be applied to higher‐order resonant modes of anisotropic nanoparticles.^[^ ^47^, ^49^ ^]^

## 5 Comparison of the Near‐Field Results to the Theory

**Figure 4:**
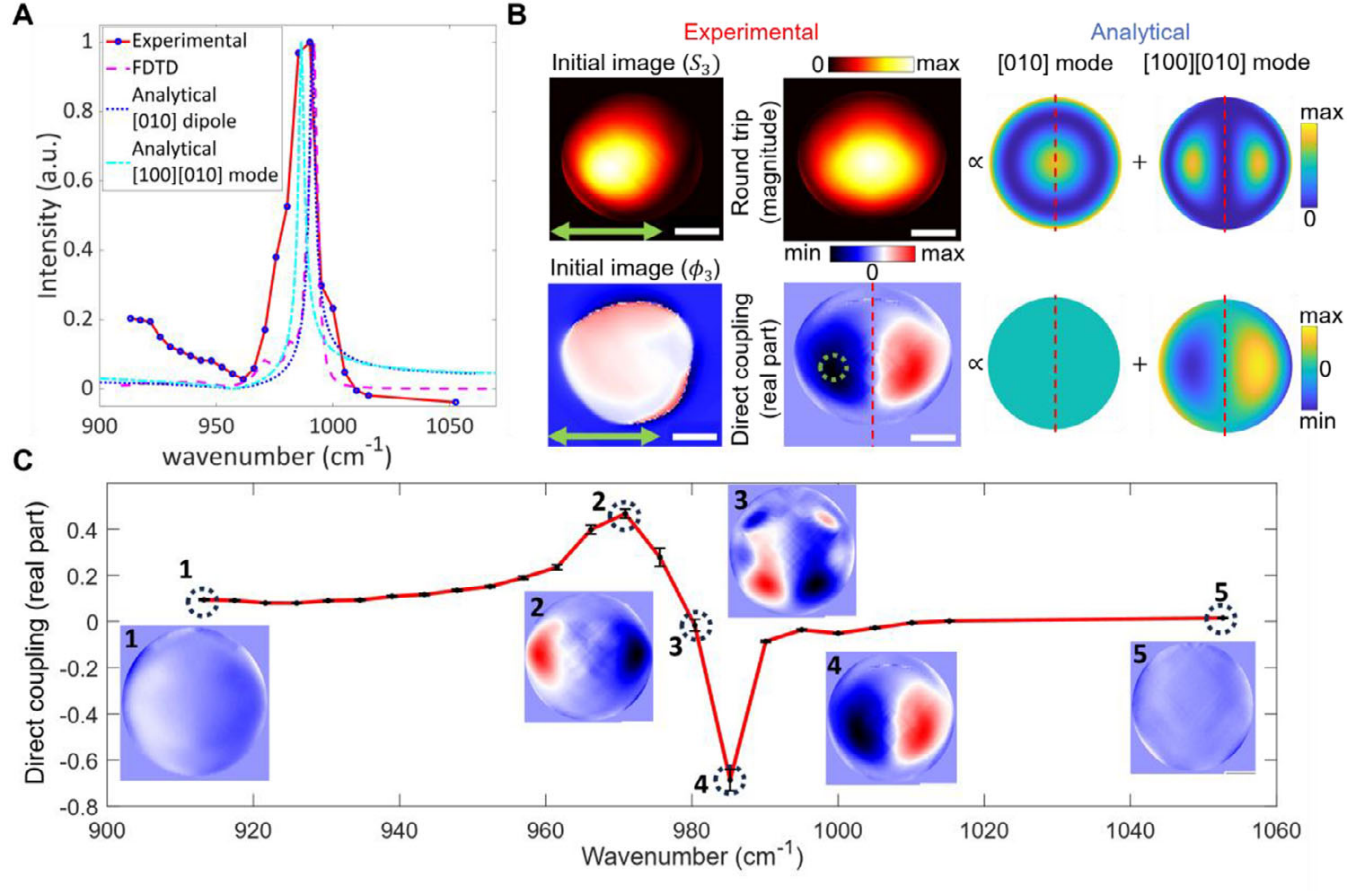
s‐SNOM measurements and analysis of the first α‐MoO_3_ oblate nanoellipsoid (***a*** = ***b*** = 112 nm, c=220 nm). A) Comparison of the measured near‐field phase intensity spectrum to the normalized absorption of the [010] dipole mode from FDTD simulation (purple dashed line) and analytical model (black dotted line) and the [100][010] second‐order mode from the analytical model. B) Analysis of the s‐SNOM signal at 985 cm^−1^ (3rd harmonic) and decomposition into the DCo and RTr contributions using the symmetry axis (red dotted line). The green arrow indicates the direction of the incident beam polarization in the image plane. The DCo (RTr) image extracted from the experiment is compared to the sum of the theoretically predicted contributions of the [010] out‐of‐plane mode and the [100][010] in‐plane modes. The white scale bar is 125 nm in size. C) Spectral measurement of the antisymmetric contribution for α‐MoO_3_ oblate nanoellipsoid averaged over the green circular area in (B), showing resonance behavior. Snapshots of the direct coupling are displayed in the graph, highlighting a distinct flip occurring after the mode's resonance.

**Figure** **4**A presents the normalized near‐field 3^rd^ harmonic phase spectra for a representative oblate nano‐ellipsoid (*a* = *b* = 56 nm, *c* = 110 nm) over the nanoparticle area (see Figure S7, Supporting Information). The spectra clearly show a resonance at 988 cm^−1^, with a Q factor of ≈138. Notably, this measured resonance frequency is blueshifted compared to the bulk α‐MoO_3_ resonance at ≈958 cm^−1[^ ^38^ ^]^ (see Figure S8, Supporting Information). In addition, a measured smaller oblate nano‐ellipsoid (*a* = *b* = 35 nm, *c* = 90 nm) exhibited an even higher Q factor of ≈ 285 (see Figure S9, Supporting Information). These values are significantly higher than those previously reported for α‐MoO_3_ nanodisks and slabs.^[^ ^28^, ^36^ ^]^

The measured spectrum is compared to the normalized dipole‐mode resonance along the [010] crystal axis and the [100][010] higher‐order mode resonance from the analytical theory (Figure 3A). We also compare our results to the absorption spectrum from the Finite‐difference Time‐Domain (FDTD, Lumerical Ansys) simulation of an α‐MoO_3_ nanoellipsoid illuminated by a plane wave with an electrical field along the [010] crystal axis. The [010] mode peaks obtained from the theoretical analysis and FDTD simulation very closely match the location of the measured near‐field peak at 988 cm^−1^. The measured near‐field amplitude spectrum of the nano‐ellipsoids also indicates a secondary resonance at ≈980 cm^−1^ (see Figure S7, Supporting Information), close to the dipole mode, in agreement with our theoretical results for the nearby high‐order [100][010] mode. Measurements on additional nano‐ellipsoids confirmed this secondary resonance (see Figure S9, Supporting Information).

Figure 4B shows the DCo and RTr contributions near the resonance peak in Figure 4A compared to the predicted DCo and RTr contributions from the out‐of‐plane [010] dipole and in‐plane [100][010] high‐order mode as predicted by the analytical theory via *E_z_* (***r***_0_) and $E_{z}^{2}\left(r_{0}\right)$ for the far‐field and near‐field contributions, respectively. The two left images show the 3^rd^ harmonic near‐field amplitude and phase image at 985 cm^−1^. The RTr and DCo images extracted from the experiment are in excellent agreement with the combined RTr of the out‐of‐plane [010] dipole and in‐plane [100][010] higher‐order mode predicted by the analytical theory. This agreement assumes a more dominant dipole mode contribution. The term ’combined RTr’ refers to the superposition of contributions from both the dipole and higher‐order modes (see Methods Section for additional explanations, and Figure S10, Supporting Information). Figure 4C illustrates the normalized spectrum of the DCo, which is an average of measurements taken within the green‐circled area in Figure 4B. Both the spectrum and the accompanying images of the real part of the direct coupling exhibit a resonance at ≈980 cm^−1^, with mode flipping (change in the sign or orientation of the mode) after the resonance peak. This behavior can be explained by considering an expression of the electric field close to a resonance of the form^[^ ^40^, ^48^, ^49^ ^]^ $E\propto \frac{1}{\varepsilon_{z}\left(\omega \right)-\varepsilon_{zm}}|E_{l,m}\rangle ,$ which shows that there is a sign change when crossing a resonance.

**Figure 5:**
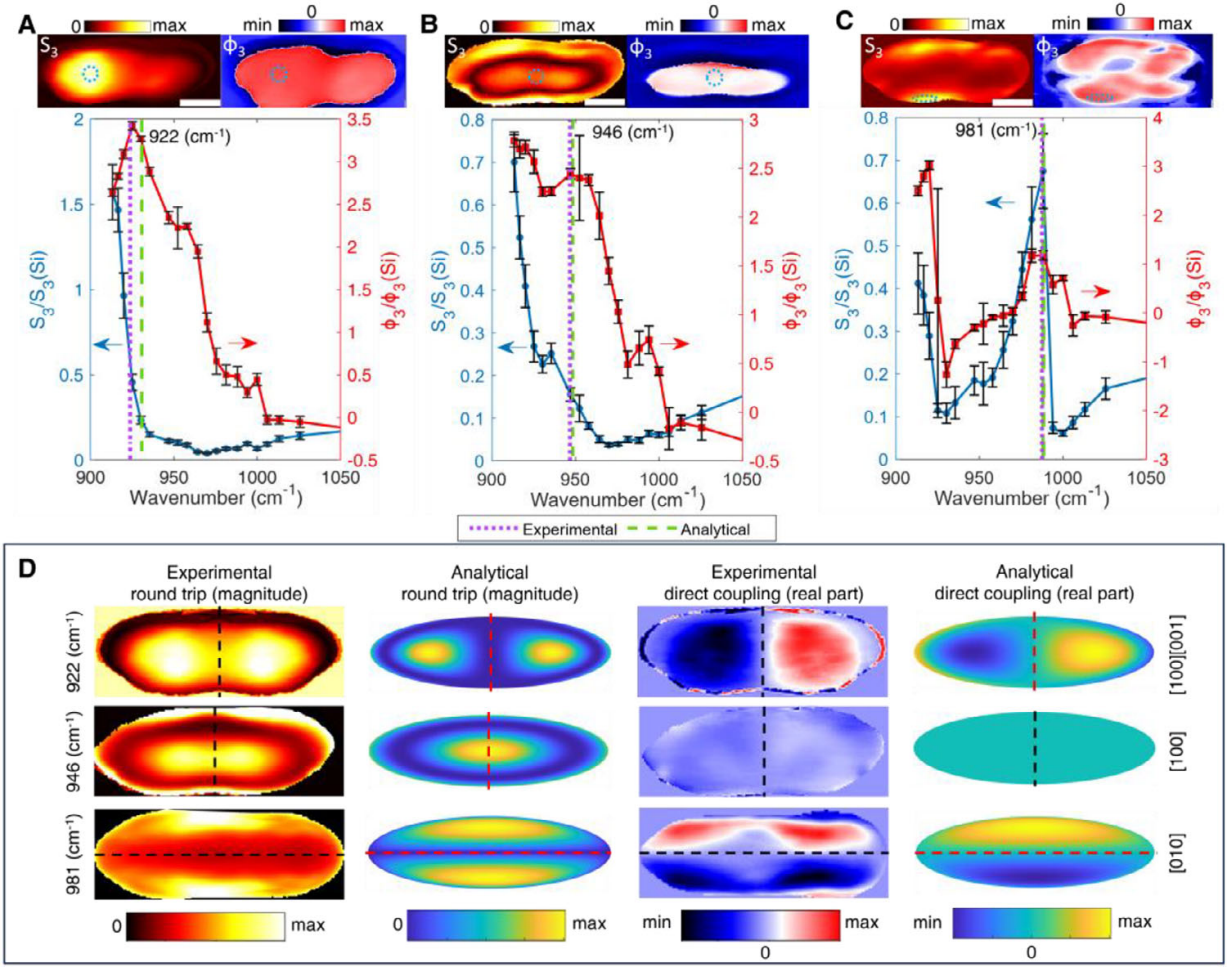
Mid‐IR s‐SNOM scan of the α‐MoO_3_ prolate ellipsoid (b=c= 60 nm, a=200 nm) and analytical model results. A) Near‐field amplitude and phase spectrum exhibiting a resonance at 925 ***cm*** ^−1^. The inset shows a near‐field amplitude and phase image of the prolate nanoellipsoid at 922 cm^−1^ with a green circle indicating the measurement area for the spectrum. The white scale bar is 150 nm size. B) Near‐field amplitude and phase spectrum showing resonance at 946 cm^−1^. The inset shows a near‐field amplitude and phase image of the nanoellipsoid at 946 cm^−1^ with a green circle indicating the measurement area for the spectrum. C) Near‐field amplitude and phase spectrum with a resonance at 981 cm^−1^. The inset shows a near‐field amplitude and phase image of the prolate ellipsoid at 981 cm^−1^. D) DCo and RTr for each measured resonance in A‐C, and the corresponding theoretical prediction for the [100][001], [100], and [010] modes, respectively (see Figure 3F).

Some synthesized α‐MoO_3_ nanoparticles have a prolate ellipsoidal shape, resulting in higher aspect ratios than the previously considered α‐MoO_3_ nanoellipsoids. **Figure** **5** shows near‐field measurements for a representative prolate nanoellipsoid with *b* = *c* = 60 nm,  *a* = 200 nm (see Figure S11, Supporting Information). Figure 5A–C presents the normalized spectra of the ellipsoid phase and amplitude at the ellipsoid's radial focus, center, and bottom edge, respectively. When examining both near‐field amplitude and phase spectra we observe three distinct resonances at ≈925, 946, and 981 cm^−1^, corresponding to a second‐order mode, an out‐of‐plane dipole mode, and a vertical dipole mode (along the short ellipsoid axis), respectively. In this ellipsoid, ε_*x*_↔ε_001_,ε_*y*_↔ε_010_,ε_*z*_↔ε_100_;we therefore expect to observe resonances associated with ε_*y*_,ε_*z*_ in our 900‐1000 cm^−1^ frequency range, see Figure 1C. Clearly, the dipole modes are blueshifted compared to the bulk resonances, since the negative eigen‐permittivities are approached at higher frequencies than the bulk resonant frequencies, where the permittivity is infinite. Moreover, as the [100] bulk peak is wider compared to the [010] peak, we expect the resonances associated with [100] to be more blueshifted from the bulk peak compared to [010]. Specifically, the exact resonance locations were calculated using the analytical expressions in the paper: ε_*z*_ = ε_*y*_ = −1.21, (60^2^ε_*x*_ + 200^2^ ε_*z*_)/(60^2^ + 200^2^) = −1.33, (ε_*y*_ +  ε_*z*_)/2 = −1.07, $\frac{\left(60^{2}\varepsilon_{x}+200^{2}\varepsilon_{y}\right)}{\left(60^{2}+200^{2}\right)}=-1.33,$ see SI for details. From this we expect the dipole resonances along the [100] and [010] axes to occur at 940 cm^−1^ and 988 cm^−1^, respectively, and the pronounced high‐order [100][001] mode peak to occur at 931 cm^−1^. These values are in very good agreement with the experimentally measured resonances. The dipole results also match the far‐field FDTD simulation of the ellipsoid dipole modes (see Figure S12, Supporting Information). Figure 5D compares the DCo and RTr images extracted from the experimental data near each resonance peak to the corresponding images calculated from the analytical theory. The experimental DCo and RTr distributions imaged at 922, 946, and 981 cm^−1^ are in very good agreement with the theoretical [100][001], [100], and [010] mode projections *on the s‐SNOM tip*, respectively. Our work showcases the remarkable capability of our α‐MoO_3_ nanoellipsoids to enable both dipole and higher‐order LPhPs across a broad MIR spectral range. Moreover, the notable variations in the spectral properties between the two measured nanoellipsoids underscore the exceptional resonance tuning capability achievable with our system.

## 6 Conclusion

We studied the optical response of α‐MoO_3_ biaxial nanoparticles experimentally for the first time. These subwavelength α‐MoO_3_ nanoparticles, synthesized using a novel method of fs‐laser pulse ablation in liquid, support directional first‐ and higher‐order LPhPs in the 8.5‐11 µm spectral range. We also developed a complete theoretical analysis of general anisotropic particles in the quasistatic regime. We obtained excellent agreement between the theoretically predicted 1^st^ and 2^nd^ order modes and the hyperspectral near‐field measurements of the spectra and mode distributions. We observed 2^nd^ order modes at frequencies even lower than the first‐order mode resonance. Moreover, in contrast to isotropic nanostructures, the resonances of high‐order modes couple the permittivities in the different axes via basic sum rules.

Our α‐MoO_3_ nanoellipsoids demonstrate pronounced resonances in the MIR range, accompanied by remarkably high Q factors. The strong confinement results in a blueshift of the LPhPs of ≈32 cm^−1^, indicating significant potential for a broad range of spectral‐response tunability. In contrast to isotropic nanoellipsoids, which can exhibit distinct resonances at separate frequencies but with a single pronounced dipole peak, our α‐MoO_3_ nanoparticles possess naturally occurring multiple‐frequency behavior. They support several dominant directional modes with comparable strengths. This multispectral nature of the biaxial particle enables imaging in at least three spectral regions. By binding a nanoparticle to a target protein, this multispectral probe can function as a biomarker with enhanced specificity of the spectral signature.^[^ ^55^ ^]^ In addition, the environment‐dependent resonance shifts of the particle can be harnessed to measure the biological environment, which may lead to enhanced cancer detection capabilities.

In summary, our findings open new avenues for IR detectors, image sensors, and photonic devices such as multispectral detectors, photonic gates, biomarkers, and directional dark‐matter detectors.^[^ ^56^, ^57^ ^]^ Our theoretical analysis also applies to ferromagnetic particles, ice grains, and liquid crystal droplets.^[^ ^22^, ^58^, ^59^, ^60^ ^]^ Finally, it is expected to apply to other fields of physics, such as quasi‐magnetostatics and heat conduction.^[^ ^61^, ^62^ ^]^

## Conflict of Interest

The authors declare no conflict of interest.

## Supporting information



Supporting Information

## Data Availability

The data that support the findings of this study are available from the corresponding author upon reasonable request.
