# Supplementary material for: Observation of Localized Resonant Phonon Polaritons in Biaxial α‐MoO3 Nanoparticles
Source: Adv Sci (Weinh). 2025 Sep 15;12(43):e17123. doi: 10.1002/advs.202417123 (PMC12631883; doi:10.1002/advs.202417123)
Supplement: Supplementary file 1 — Supporting Information [file ADVS-12-e17123-s001.docx]

Supplementary Materials for

**Observation of Localized Resonant Phonon Polaritons in Biaxial α-MoO3 Nanoparticles**

**Authors:** Daniel Beitner^1,2,3^*†, Asaf Farhi^3^†, Ravindra Kumar Nitharwal^4^, Tejendra Dixit^5^, Tzvia Beitner^1^, Shachar Richter^1,2^, SivaRama Krishnan^4^ and Haim Suchowski^2,3^

**Affiliations:**

^1^Department of Materials Science and Engineering, Faculty of Engineering, Tel Aviv University; Tel Aviv, 6997801, Israel.

^2^University Centre for Nanoscience and Nanotechnology, Tel Aviv University; Tel Aviv, 6997801, Israel.

^3^School of Physics and Astronomy, Faculty of Exact Sciences, Tel Aviv University; Tel Aviv, 6997801, Israel.

^4^Department of Physics and Quantum Center of Excellence for Diamond and Emerging Materials, Indian Institute of Technology Madras; Chennai, 600036, India.

^5^Department of Electronics and Communication Engineering, Indian Institute of Information Technology Design and Manufacturing (IIITDM); Kancheepuram, 600127 India.

*Corresponding author. Email: Beitner@mail.tau.ac.il

† These authors contributed equally to this work

Corresponding author: Daniel Beitner, beitner@mail.tau.ac.il

**The PDF file includes:**

Materials and Methods

Supplementary Text

Figs. S1 to S12

References

Materials and Methods

α-MoO_3_ nanoparticles synthesis:

MoO_3_ pellets were used as ablation targets in the femtosecond pulsed laser ablation in a liquid (fs-PLAL) process. The pellets were made from powder (Sigma-Aldrich, USA) through powder grinding and palletization using a hard press at a pressure of 5 MPa. The resulting pellets were then sintered at 560 ͦ C for 12 hours. X-ray diffraction (XRD) and Raman spectroscopy were used to analyze the pellets and confirm the formation of the α-MoO_3_ phase. The ablation process was carried out using a Ti:Sapphire femtosecond laser (Astrella, Coherent Inc., Santa Clara, CA, USA) with a central wavelength of 800 nm, repetition rate of 1 kHz, full width at half-maximum of 500 fs, and an average power of 1.5 W. The laser beam was focused on the target using a converging lens with a focal length of 20 cm. A pre-prepared α-MoO_3_ pellet was placed at the base of a beaker filled with 30 mL of acetone (Sigma-Aldrich).

Acetone was chosen as the solvent because of its inherent stability, which helps to protect the pellet in its liquid environment. A fluid layer above the α-MoO_3_ particle prevents exposure to ambient air. The Rayleigh range of the ablating femtosecond laser beam must be entirely within the solvent layer above the sample for proper beam focusing. To minimize the potential impact of the laser on the targeted area, the pellet enclosed in the glass beaker was continuously moved using a translation stage. The translation stage can move in two dimensions and is operated by an automated program. The ablation procedures were conducted for 20 minutes. Afterward, the solution of nanoparticles (NPs) was carefully stored in hermetically sealed containers. Subsequently, the solution was transferred to centrifuge tubes to facilitate debris separation. Centrifugation was performed at a rotational speed of 5000 revolutions per minute (RPM) for 30 minutes. After centrifugation, unwanted debris was precipitated in the lower region of the tube, while the top portion consisted exclusively of NPs. A pipette was used to isolate these NPs from any accompanying debris. The addition of acetone further diluted the resulting NP/acetone solution. Samples for scattering-scanning near-field microscope (s-SNOM) measurements were made by drop-casting onto a clean Si prime wafer and dried at room temperature. The NP size and shape were measured using a combination of AFM for height measurement and scanning electron microscopy for measurements of the lateral dimensions.

Near-Field Imaging:

Near-field and AFM measurements were taken using a commercial s-SNOM (Neaspec Attocube). The system uses a Pt-coated AFM probe (PPP-NCLpt from NANOSENSORS) to scan the sample in tapping mode (160 kHz frequency, 60 nm amplitude). A tunable Mid IR (910 – 1205 cm^-1^) QCL (Mircat DRS daylight solutions) operating at 3 mW is used for the optical near-field measurements. All the results were taken from the 3^rd^ demodulation harmonic order of the probe tapping frequency to reduce far-field noise in the signal. Furthermore, a pseudo-heterodyne measurement system extracts the optical phase and further reduces far-field noise in the measurements. Localized spectra were taken using point spectroscopy^1^. Consecutive scans of the sample were taken at different wavelengths and combined using a dedicated Python script^1,2^ (correcting for the position of the particle and drift in each scan) to compose a high-resolution hyperspectral scan of the sample. The reported spectra are normalized with respect to the spectra of the Si substrate. Phase images were corrected for thermal drift of the interferometer reference arm using the Gwyddion ^3^ line scan correction algorithm. The s-SNOM is used to measure the near-field signal of a resonating structure, and it can be decomposed into four distinct scattering processes that are possible for a two-particle system. It is important to note that all these processes must involve coupling to the probe; otherwise, they would be filtered out during demodulation. These contributions are: ^4^

1. Signals arising from material contrast due to local polarizability of the sample, which is directly measured via the probe.
2. The round-trip signal is generated by resonant modes induced in the sample by the AFM tip, which then re-interact with the probe to scatter into the far field. As the coupling is exclusively through the AFM tip, both dark and bright modes can be excited regardless of their orientation with respect to the incident-beam polarization. Round-trip represents the sum of the magnitudes of the different resonant modes in complex image data.
3. The direct coupling signal is of the incident beam that directly excites the sample, scattered to the AFM tip and then scattered to the far-field. It is worth noting that this contribution also includes the opposite path of the tip exciting a resonant mode, which is then directly measured by the detector. The opposite path contribution is included in the signal as they are identical in reciprocal samples. Direct coupling is a good representation of the *z*‑component of the electric field of the resonant mode and, as such, is displayed as the real part of the complex image data.
4. Modulated scattering occurs when the incident beam is directly coupled in and out of the resonant structure but is perturbed by the presence of the probe near the structure, thus modulating the signal. Generally, this contribution is negligible for single-particle samples ^4^.

Of these four processes, only b and c have been shown to contribute significantly to imaging NP modes^4^. Using the different symmetry properties of these contributions, we decompose the measured field to the RTr and DCo contributions. The RTr contribution is always positive since it is proportional to the square of the $z$ field component of the **resonant** **mode** $\left| E_{z}(r) \right|^{2}$ ^4^, see SI p. 15-18. The DCo contribution is proportional to $E_{z}(r)$ and it typically changes sign along the image. For the case of symmetrical NP geometries with a crystal axis aligned with the geometrical axis of the NP (such as nano-ellipsoids where the different crystal axis are aligned with the ellipsoid in other radial directions), we expect for in plane modes RTt to have an even symmetry across the crystal axis and DCo to have the odd symmetry of the mode. Since our particles are anisotropic, the modes are directional along the crystal axes, and the symmetry is with respect to them, unlike the analysis in Ref. ^4^. Thus, by using DCo odd symmetry properties, we can separate its contribution to the complex near-field signal $\sigma_{n}$ by subtracting its replica mirrored across the odd symmetry axis $\sigma_{n,\text{mirror}}$ to get $\sigma_{DCo}={\frac{1}{2}(\sigma}_{n}-\sigma_{n,\text{mirror}})$, and the RTr image can be generated by adding the mirrored signal $\sigma_{RTr}={\frac{1}{2}(\sigma}_{n}+\sigma_{n,\text{mirror}})$.

In certain situations, both RTr and DCo will be symmetrical and appear identical in the s-SNOM scan, such as when the mode is completely oriented along the out of plane (the AFM prob length direction). As out of plane mode have identical RTr and DCo contribution we can extract the mode shape directly from the s-SNOM scan data without separating to symmetric and anti-symmetric contributions. Thus, we have three different procedures when analyzing the data depending on if there are out of plane modes, in-plane modes or a combination of the two:

Table 1 – Procedure to extract mode shape from s-SNOM scan data.

| Out-of-plane mode only | Both DCo and RTr shape will be symmetrical in the scan, and they will be similarly shaped. symmetrical signal will then contain both. |
| --- | --- |
| In plane modes only | We separate symmetric (RTr) and antisymmetric (DCo) components perpendicular to the mode direction |
| Combination of in-plane and out-of-plane modes | We separate symmetric (RTr) and antisymmetric (DCo) components perpendicular to the in-plane mode direction. The in-plane mode shape can be taken from the antisymmetric (DCo), as out-of-plane modes are symmetrical. The symmetric (RTr) is a combination of both mode shapes. |

While the main excitation of high-order modes is from the tip, which behaves as a point dipole, the far field can excite the higher-order modes with lower intensity, which can be approximated by $\left( \frac{2a}{\lambda} \right)^{n-1}\approx\left( \frac{1}{8} \right)^{n-1}$, where *n* is the mode order, and $a$ is the particle size. At the same time, sharp interfaces between inclusions are known to generate scattered fields with short wavelengths, and hence, in our setup, the substrate-particle interface may result in the excitation of high-order modes. An additional mechanism to couple a far field to high-order modes is the coupling between the crystal modes $k\to k+mg$, where $g$ is the crystal wavevector, which decreases for large integer $m$, see Refs^5–7^.

Near-Field Data Analysis:

The data from the s-SNOM measurements was analyzed using a combination of Gwyddion ^3^ and Python script. This was especially needed for analyzing the data from the multiple scans used for the hyperspectral data. The hyperspectral data was taken from multiple consecutive scans of the chosen imaging area, while the QCL wavelength was scanned across its spectral range. To extract a normalized near-field spectrum, we first correct each scan using Gwyddion, and then, using the Python script, we combine the data. Phase scans are corrected for thermal drift of the interferometer scan, while amplitude data is taken as is. Gwyddion correction uses the topological z-data from the AFM scan of the s-SNOM to mask areas of the scan as the Si background. The baseline of z-data and the phase data are corrected using the following two steps applied to the Si area map data:

- Removal of polynomial background: on the vertical direction with a polynomial degree of 2
- Aligning rows using the matching method

A Python script uses the z-data to locate the particle boundary in the scan via the change in height. The script then shifts the scan data to the center of the particle. Furthermore, the thermal drift of the AFM on the slow scan axis is corrected by adding a linear correction to the data. The data for each scan is normalized ($S_{\mathrm{sample}}/ S_{\mathrm{Si}}$and$\Phi_{\mathrm{sample}}-\Phi_{\mathrm{Si}}$) using the measured signal for the Si substrate as it is constant in the MIR range. All the data is taken from the third harmonic of the demodulated signal of the s-SNOM.

FDTD modeling:

The FDTD was performed using Lumerical software from Ansys. The ellipsoid was modeled using dielectric data taken from ^8^. The system was modeled as an ellipsoid on a semi-infinite Si substrate with a broadband Total-Field Scattered-Field (TFSF) source. The source is a pulsed plane wave propagating in the *z*-axis with polarization along the *x*-axis. The crystal axis of the sphere is defined with respect to the direction of the source polarization. Near-field images were taken from a monitor tangent to the sphere and perpendicular to the source propagation direction (in the *x-y* plane). This enables us to simulate the NP’s far-field interaction and image the real component of the *z*-aligned electric field on the top of the NP. The *z*-aligned electric field correlates with the direct coupling component of the near field. The simulation ran for 15000 fs to allow full decay of all residual fields. The mesh size inside the source was set to 2.5 nm.

The oblate α-MoO_3_ nano-ellipsoids were simulated as a [$r_{x},r_{y},r_{z}] =$[110,110,55] nm ellipsoids with crystal axes aligned as follows: [010] – along the *z*-axis, [100] and [001] – are both pointed in the *x-y* plane.

The α-MoO_3_ prolate ellipsoid was simulated as an ellipsoid with a major axis of 200 nm and minor axes of 60 nm. The crystal axes are aligned as follows: [001] – along the long axis of the ellipsoids, which matches previously reported growth of MoO_3_ rod-like shaped particles ^9,10^.

Supplementary Information

**S1:Further Information on the α–MoO_3_ NPs synthesis**

Femtosecond pulsed laser ablation in a liquid (fs-PLAL) is a novel and evolving method for synthesizing *α*-MoO_3_ NPs, enabling the production of particles of varying sizes. The *α* -MoO_3_ pellet is an ablation target inside the liquid medium to synthesize the NPs. The structural analysis of the synthesized NPs was conducted using X-ray diffraction (XRD) technique, as seen in Fig. 1B. X-ray diffraction (XRD) using Cu Kα radiation (λ = 1.5405 Å) is employed to get details of the crystal phase and structure of the α-MoO_3_ pellet as well as synthesized α-MoO_3_ NPs. The scan range was set to 5 to 80^o^ at a scan rate of 0.068 o/s. In the XRD pattern of the α-MoO_3_ pellet (Fig 1a), all the diffraction peaks show good agreement with the JCPDS 05-0508 (space group Pbnm (62) and unit cell parameters a = 3.96 Å, b = 13.86 Å, and c = 3.7 Å) and Miller indices are specified accordingly^11–13^. The observed intensities of reflection peaks of (020), (040), and (060) are very strong in the XRD pattern of α-MoO_3_ pellet and are the characteristic diffraction peaks of the α-MoO_3_ orthorhombic system ^11–16^.


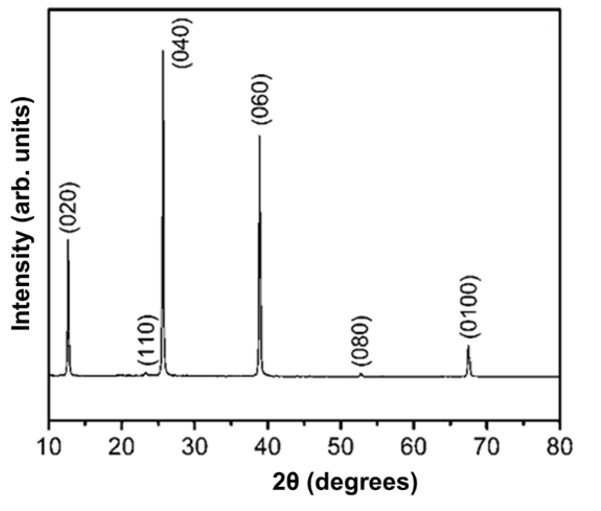


**Fig. S1. XRD pattern on the crystalline α-MoO3 nanostructures (adapted from Ref** ^17^**).**

The XRD pattern of the synthesized α-MoO_3_ NPS displays intense lattice planes (020), (040), and (060) similar to Ref ^17^, confirming the formation of crystalline α-MoO_3_ NPs with preferential orientation in [010] direction.^17^

Dewangan et al. synthesized the α-MoO3 crystalline nanostructures with the preferential orientation along the [010] direction, as shown in Fig. S1.^17^ In their study, the XRD pattern shows stronger intensities of (020), (040), and (060) planes, indicating the highly anisotropic growth as well as the preferred orientation of the crystalline α-MoO_3_ nanostructure in [010] direction ^17^. In our case, the XRD pattern of the α-MoO_3_ NPs drop cast on the Si substrate (Fig 1a) shows a similar diffraction pattern with highly intense atomic planes (020), (040), and (060) compared to others, representing the anisotropic growth in the [010] direction and evidencing the existence of the lamellar structure of crystalline α-MoO_3_ NPs^16–18^. The other low-intensity XRD peaks observed in the α-MoO_3_ pellet are not found in the α-MoO_3_ NPs, suggesting the formation of the highly crystalline orthorhombic phase of α-MoO3 NPs with preferred orientation only in the [010] direction^17,18^. In addition, the diffraction peaks (220) and (400) in the XRD of α-MoO_3_ NPs belong to the silicon (Si) substrate (JCPDS: 27-1402). Interestingly, no peaks of any other phases or impurities were detected, indicating high purity of the α-MoO_3_ NPs. Consequently, XRD results prove the pure crystallinity in the form of orthorhombic phase α-MoO_3_ NPs. Furthermore, the elemental composition of the synthesized α-MoO_3_ NPs was determined by energy-dispersive X-ray (EDX) spectroscopy. The EDX spectra of synthesized α-MoO_3_ NPs on carbon tape shown in Fig. S2 confirm the presence of molybdenum (Mo) and oxygen (O) atoms, which agree with the reported work on EDX of α-MoO_3_ NPs. ^19–21^. No other elements were detected, which confirmed the purity of the α-MoO_3_ NPs.


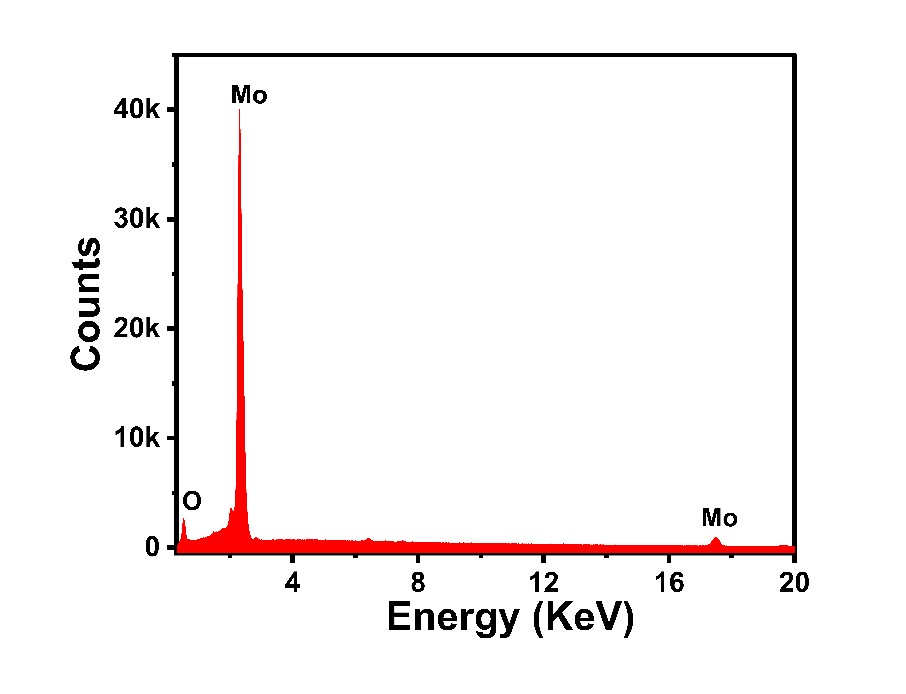


**Fig. S2. Energy-dispersive X-ray (EDX) spectra of the synthesized α-MoO3 NPs.**

The inspect F50 (FEI Company USA) system’s field emission scanning electron microscope (FESEM) is used to characterize the surface morphology of the synthesized NPs, as seen in Fig. S3A.

The vibrational spectra of the synthesized α-MoO3 NPs were determined by Raman spectroscopy as shown in Fig. S3B. Raman spectra of α-MoO_3_ pellet and NPs were measured in the 100‑1000 cm^-1^ range using a Horiba Jobin Yvon HR800 UV spectrometer with a 532 nm wavelength laser as an excitation source and spot size of ~1 μm. The highest resolution grating, 1800 lines/mm with a resolution of ~ 1 cm^-1^, was used during measurement. The Raman spectra of the α-MoO_3_ pellet and synthesized NPs (drop cast on a Si substrate) are shown in Fig. S3B bottom and top, respectively. The Raman modes at 995 cm^-1^, 820 cm^-1^, and 665 cm^-1^ are the stretching modes of different oxygens. The stretching mode at 995 cm^-1^ (Ag, B1g) corresponds to the antisymmetric stretch of the terminal oxygens, while the stretching mode at 820 cm^-1^ (Ag, B1g) is the symmetric stretch of terminal oxygens. The mode at 665 cm^-1^ (B2g, B3g) comes from the antisymmetric stretching of the Mo-O-Mo bridges along the c-axis. Other Raman modes are also observed, such as a mode at 471 cm^-1^ (Ag, B1g, O-Mo-O stretch, and bend), two scissors modes at 378 cm^-1^ (B1g), and modes at 365 cm^-1^ (Ag), 336 cm^-1^ (Ag, B1g, δ O-M-O bend), 293 cm^-1^ (B3g, δ O=M=O, wagging), 285 cm^-1^ ( δ O=M=O, wagging), 247 cm^-1^ (B3g, τ O=M=O, twist), 216 cm^-1^ (Ag, rotational rigid MoO_4_ chain mode, Rc), 197 cm^-1^ ( B2g, τ O=M=O, twist), 159 cm^-1^ (Ag, B1g, translational rigid chain mode, Tb), 129 cm^-1^ (B3g, translational rigid MoO4 chain mode, Tc), 98 cm^-1^ (B2g, translational rigid MoO_4_ chain mode, Tc) ^22,23^.


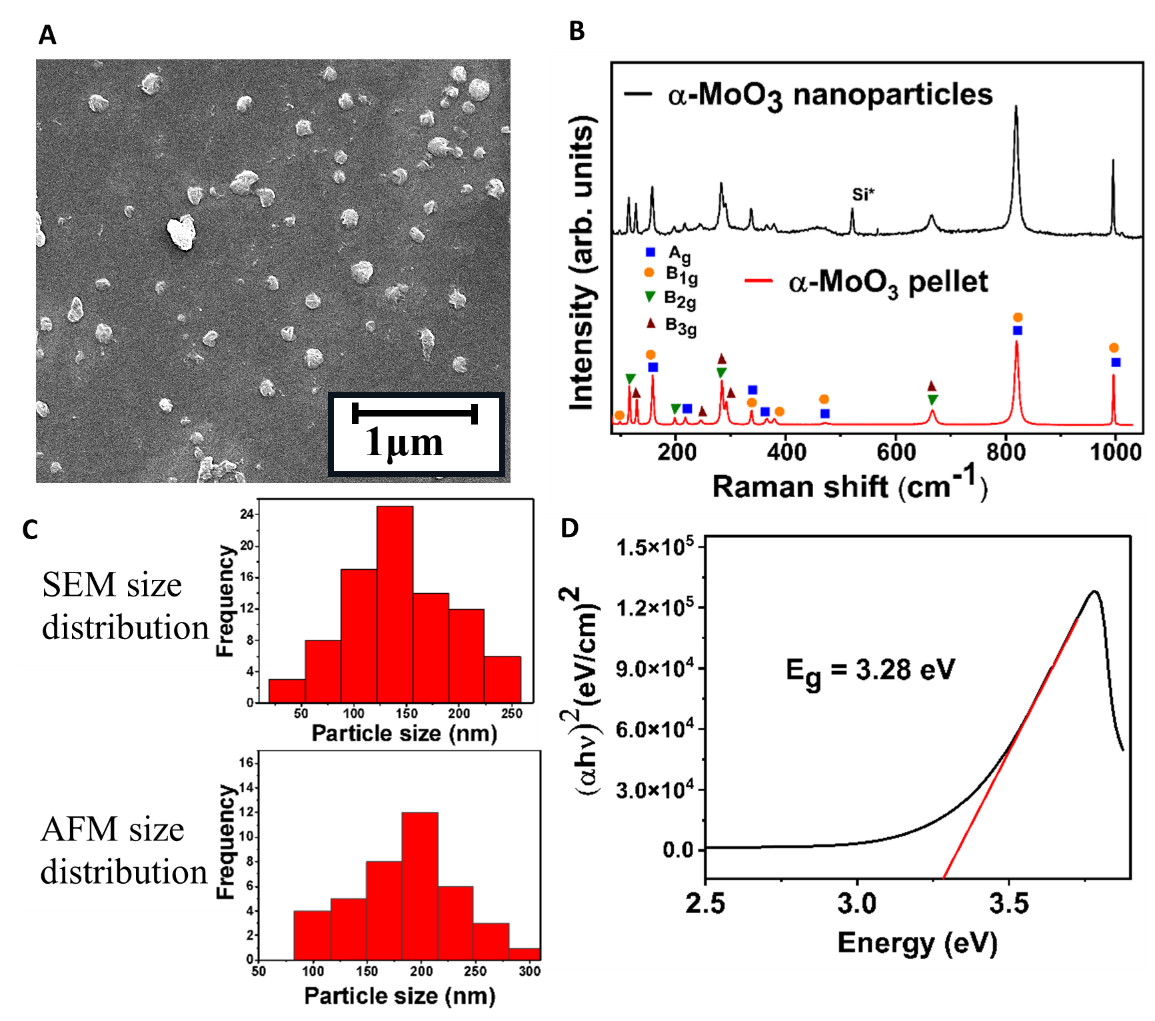


**Fig. S3.** **Analysis of morphology and size of fs-PLAL α-MoO_3_ made NPs**. (**A**) (define FE) FESEM image of the synthesized NPs. (**B**) Room temperature Raman spectra of α-MoO_3_ pellet and collected NPs with a 532 nm excitation laser. Different colour symbols denote the Raman active modes in the Raman spectra of α-MoO_3_, while the Raman mode at 521.5 cm^-1^ is the silicon substrate mode. (**C**) Size distribution of α-MoO_3_ NPs as measured via SEM and AFM on 10x10 $\left( \mu m \right)^{2}$ images. (**D**) Tauc plot of MoO_3_ NPs with the direct bandgap of 3.28 eV.

The calculated average particle long diameter is between 100-200 nm, confirmed by the atomic force microscope (AFM) image, as shown in Fig. S3C above. These synthesized NPs show a direct band gap nature, estimated by the Tauc plot as shown in Fig. Fig. S3D above.

**S2 – Electro-quasistatic analysis of biaxial spheres and ellipsoids**

To calculate the eigenstates in the quasistatic regime, in which the typical length scale is much smaller than the wavelength, one can solve Laplace’s equation without a source, which reads for anisotropic media:

$\nabla\cdot\epsilon\nabla\psi_{n}=0,$ (S2.1)

where $\psi_{n}$ is an eigenstate, which is an electric potential that exists without a source for certain values of the eigenpermittivities $\epsilon=\epsilon_{1n}$ inside the inclusion and $\epsilon=I\epsilon_{2}$ otherwise, where $I$ is the identity matrix. Clearly, $\boldsymbol{E}_{n}=\nabla\psi_{n}$ have to satisfy the boundary conditions at the particle interface. Analyzing the response of anisotropic inclusions entails several challenges: the symmetry of the material/crystal may differ from the symmetry of the inclusion, which makes it hard to formulate the problem, $\epsilon_{1i}\neq\epsilon_{1j}$must be satisfied, unlike for isotropic inclusions, and as opposed to standard eigenvalue problems with a single degree of freedom, there are several degrees of freedom of the axial permittivities. One can readily see that the widely employed spherical harmonics cannot typically be eigenstates of anisotropic spherical particles.

Here, we analyze the eigenmodes and eigenpermittivities of anisotropic spheres and ellipsoids. We start with the somewhat known dipole response and proceed to the unexplored high-order mode response. The anisotropic dipole eigenstates and eigenpermittivities of a sphere and ellipsoid can be deduced from the scattering analyses in the literature and read for a mode oriented along $z$:

$$\tilde{\psi}_{sphere, l=1,m=0}=\frac{1}{\sqrt{a}}\left\{ \begin{matrix} \frac{r}{a}\text{cos}\theta& r<a \\ \frac{a^{2}}{r^{2}}\text{cos}\theta& r\geq a \end{matrix},\epsilon_{1z}=-2, \right.$$

$\tilde{\psi}_{l=1,m=0,inside ellipsoid}\propto z,\epsilon_{1z}=\epsilon_{2}(L_{z}-1)/L_{z},$ (S2.2)

$$L_{z}=\frac{abc}{2}\int_{0}^{\infty} \frac{dq}{\left( c^{2}+q \right)R\left( q \right)}, {R\left( q \right)\equiv\left[ \left( q+a^{2} \right)\left( q+b^{2} \right)\left( q+c^{2} \right) \right]}^{1/2}$$

where in the case of an ellipsoid $a,b,c$ are the intersections of ellipsoid with the positive $x,y,z$ axes, respectively. While these eigenstates and eigenpermittivities appear to be identical to the isotropic ones, here, the modes are oriented along the resonant crystal axis independently of the incoming field polarization even though the direction of polarization does affect the excitation magnitude.

We now proceed to the high-order modes. Our goal is to express the eigenstates in cartesian coordinates due to the crystal symmetry and obtain relations (by satisfying boundary conditions + Laplace’s equation) that are relevant to an anisotropic medium, i.e., ones that enable $\epsilon_{i}\neq\epsilon_{j}$. Since the field outside the sphere can be expanded in spherical harmonics, the high-order spherical harmonics are suitable only for isotropic medium, and there is a continuity of the potential on the particle envelope, we conclude that the anisotropic modes are specific combinations of spherical harmonics.

We suggest the following first-second-order anisotropic mode $\tilde{\psi}_{2}^{1}= \psi_{2}^{0}+ (\psi_{2}^{2}+ \psi_{2}^{-2})/ 2,$where $\psi_{i}^{j}$ are the spherical harmonics. Inside the sphere, we write:

$\psi_{2}^{0}=\frac{1}{\sqrt{2a}}\frac{1}{a^{2}}r^{2}(3\text{cos}^{2}\theta-1)=\frac{1}{\sqrt{2a}a^{2}}(3z^{2}-r^{2}),$

$$\frac{\psi_{2}^{2}+\psi_{2}^{-2}}{2}=\frac{1}{\sqrt{2a}a^{2}}r^{2}\text{sin}^{2}\theta\text{cos}2\phi=\frac{1}{\sqrt{2a}a^{2}}r^{2}\text{sin}^{2}\theta\text{cos}2\phi$$

$=\frac{1}{\sqrt{2a}a^{2}}r^{2}\text{sin}^{2}\theta(\text{cos}^{2}\phi-\text{sin}^{2}\phi)=\frac{1}{\sqrt{2a}a^{2}}(x^{2}-y^{2}),$ (S2.3)

$$\tilde{\psi}_{2}^{1}=\frac{1}{\sqrt{2a}a^{2}}(3z^{2}-r^{2}+x^{2}-y^{2})=\frac{1}{\sqrt{2a}a^{2}}(2z^{2}-2y^{2}),$$

$${\tilde{\boldsymbol{E}}}_{2}^{1}=\frac{1}{\sqrt{2a}a^{2}}(4z\hat{z}-4y\hat{y}).$$

Imposing Laplace’s equation, we get

$\nabla\cdot\epsilon_{1}{\tilde{\boldsymbol{E}}}_{2}^{1}=4\epsilon_{1z}-4\epsilon_{1y}=0,$ (S2.4)

$$\epsilon_{1z}=\epsilon_{1y}.$$

$\boldsymbol{E}_{\parallel}$ is continuous at the interface by construction, and we impose continuity of $D_{r}.$ We first write $D_{r}$ inside the sphere:

${\tilde{\boldsymbol{D}}}_{2}^{1}=\frac{1}{\sqrt{2a}a^{2}}\left( 4z\epsilon_{y}\hat{z}-4y\epsilon_{y}\hat{y} \right),$(S2.5)

$$\tilde{D}_{2r}^{1}=\frac{1}{\sqrt{2a}a^{2}}(4z\epsilon_{y}\hat{z}-4y\epsilon_{y}\hat{y})(0,\text{sin}\theta\text{sin}\phi,\text{cos}\theta)=\frac{1}{\sqrt{2a}a^{2}}\frac{4}{r}\epsilon_{y}(z^{2}-y^{2}),$$

where we have used $\epsilon_{1z}=\epsilon_{1y}.$Note that the dot product with $r$ retrieved $y,z$ that were removed in the differentiation, which gives a factor of 2 as in the isotropic case, only that there is a freedom of choice of $\epsilon_{1x}.$

We write $\tilde{D}_{2r}^{1}$ outside the sphere in the standard way:

$$\tilde{D}_{2r,\mathrm{ext}}^{1}=\epsilon_{2}\frac{\partial\tilde{\psi}_{2}^{1}}{\partial r}=\frac{1}{\sqrt{2a}}\frac{\partial}{\partial r}\epsilon_{2}\left( \frac{a^{3}}{r^{3}} \right)2\left( \text{cos}^{2}\theta-\text{sin}^{2}\theta\text{sin}^{2}\phi\right)$$

$=-3\epsilon_{2}\frac{1}{\sqrt{2a}}\left( \frac{a^{3}}{r^{4}} \right)2(\text{cos}^{2}\theta-\text{sin}^{2}\theta\text{sin}^{2}\phi)=-3\epsilon_{2}\frac{1}{\sqrt{2a}}\left( \frac{a^{3}}{r^{6}} \right)2(z^{2}-y^{2}),$ (S2.6)

By equating them, we obtain $\epsilon_{1y}=\epsilon_{1z}=-1.5\epsilon_{2},$ which relates to a uniaxial medium inside the sphere. We now suggest the second second-order mode for an anisotropic sphere:

$$\tilde{\psi}_{2,r\leq a}^{2}=\frac{1}{\sqrt{2a}a^{2}}(\psi_{2}^{1}-\psi_{2}^{-1})/2=\frac{1}{\sqrt{2a}a^{2}}r\text{cos}\theta r\text{sin}\theta\text{cos}\phi=\frac{1}{\sqrt{2a}a^{2}}xz,$$

$${\tilde{\boldsymbol{E}}}_{2,r\leq a}^{2}=\frac{1}{\sqrt{2a}a^{2}}(z\hat{x}+x\hat{z}),$$

$${\tilde{\boldsymbol{D}}}_{2,r\leq a}^{2}=\frac{1}{\sqrt{2a}a^{2}}(z\epsilon_{x}\hat{x}+x\epsilon_{z}\hat{z}),$$

$\nabla\cdot{\tilde{\boldsymbol{D}}}_{2}^{2}=0,$ (S2.7)

$$\tilde{D}_{2r,r\leq a}^{2}=\frac{1}{\sqrt{2a}a^{2}}(z\epsilon_{x}x+xz\epsilon_{z})/r=\frac{1}{\sqrt{2a}a^{2}}zx(\epsilon_{x}+\epsilon_{z})/r,$$

$$\tilde{D}_{2r \mathrm{ext}}^{2}=\frac{a^{3}}{\sqrt{2a}}\epsilon_{2}(-3\frac{1}{r^{4}}\text{cos}\theta\text{sin}\theta\text{cos}\phi)=\frac{a^{3}}{\sqrt{2a}}\epsilon_{2}(-3\frac{1}{r^{6}}xz).$$

Thus, we obtain:

$\epsilon_{1x}+\epsilon_{1z}=-3\epsilon_{2},$ (S2.8)

which for $\epsilon_{2}=1$reads

$\epsilon_{1x}+\epsilon_{1z}=-3.$ (S2.9)

Since the choice of a system of coordinates is arbitrary, this relation should hold for any two axes. This is an interesting eigenpermittivity sum rule, this time for a general biaxial medium. In addition, there is a degeneracy for $\epsilon_{1y}=\epsilon_{1z}=-1.5\epsilon_{2}$ since both anisotropic modes are excited.

In analogy, we consider the second-order modes for anisotropic ellipsoids. We utilize the anisotropic sphere eigenstates inside the anisotropic ellipsoid, similarly to the isotropic case in Ref. ^24^. To impose continuity of $D_{r}$, we project $\boldsymbol{D}$ in the direction perpendicular to the interface of the ellipsoid:

$v_{\perp}=\left( \frac{x}{a^{2}},\frac{y}{b^{2}},\frac{z}{c^{2}} \right)/|n|, |n|=\sqrt{\left( \frac{x}{a^{2}} \right)^{2}+\left( \frac{y}{b^{2}} \right)^{2},\left( \frac{z}{c^{2}} \right)^{2}}$ (S2.10)

$$\tilde{D}_{2\perp\text{ins aniso}}^{2}=\left( zx\epsilon_{x}\frac{1}{a^{2}}+xz\frac{1}{c^{2}}\epsilon_{z} \right)/|n|=zx\left( \frac{1}{a^{2}}\epsilon_{x}+\frac{1}{c^{2}}\epsilon_{z} \right)/|n|=D_{\mathrm{ext},\perp},$$

where $v_{\perp}$is the unit vector perpendicular to the interface.

Comparing $D_{r}$ inside the ellipsoid to the isotropic case

$\tilde{D}_{2\perp\text{ins iso}}^{2}=\left( zx\epsilon_{1,xz \text{iso}}\frac{1}{a^{2}}+xz\frac{1}{c^{2}}\epsilon_{1,xz \text{iso}} \right)/|n|$ $,$ (S2.11)

we obtain that since the host medium is isotropic in both cases $D_{\mathrm{ext}}$ can be chosen to be the same in both cases, and we can impose $\tilde{D}_{2\perp ins aniso}^{2}=\tilde{D}_{2\perp ins iso}^{2}$ to satisfy the boundary condition for the anisotropic ellipsoid:

$$\frac{1}{a^{2}}\epsilon_{x}+ \frac{1}{c^{2}}\epsilon_{z}= \epsilon_{1,xz \text{iso}}\left( \frac{1}{a^{2}}+\frac{1}{c^{2}} \right)$$

where^24^

$\epsilon_{1,xz \text{iso}}=1-{((\frac{a_{x}a_{y}a_{z}}{2})(a_{x}^{2}+a_{z}^{2})I_{xz})}^{-1},$ (S2.12)

$$I_{\alpha\beta}=\int_{0}^{\infty} \frac{du}{\left( u+a_{\alpha}^{2} \right)\left( u+a_{\beta}^{2} \right)R\left( u \right)}.$$

We calculate the eigenpermittivities and eigenpermittivity sums for the oblate ellipsoid with $a= b=110\mathrm{nm},c=55\mathrm{nm},$and get:

$$\epsilon_{1a}=\epsilon_{1b}=-3.23,\epsilon_{1c}=-0.89,$$

$$\epsilon_{1xz \text{iso}}=-1.06, \epsilon_{1yz \text{iso}}=-1.06, \epsilon_{1xy \text{iso}}=-2.26$$

$$\frac{1}{a^{2}}\epsilon_{x}+ \frac{1}{c^{2}}\epsilon_{z}= \epsilon_{1,xz \text{iso}}\left( \frac{1}{a^{2}}+\frac{1}{c^{2}} \right),$$

,

$$\frac{1}{b^{2}}\epsilon_{y}+ \frac{1}{c^{2}}\epsilon_{z}= \epsilon_{1,yz \text{iso}}\left( \frac{1}{b^{2}}+\frac{1}{c^{2}} \right),$$

$$\frac{1}{a^{2}}\epsilon_{x}+ \frac{1}{b^{2}}\epsilon_{y}= \epsilon_{1,xy \text{iso}}\left( \frac{1}{a^{2}}+\frac{1}{b^{2}} \right),$$

We then perform these calculations for the prolate ellipsoid with $a=200\mathrm{nm},b=c=60\mathrm{nm}$ and obtain:

$\epsilon_{1a}=-9.48,$ $\epsilon_{1b}=\epsilon_{1c}=-1.21,$

$\epsilon_{1xz \mathrm{iso}}=-1.33,$ $\epsilon_{1yz \mathrm{iso}}=-1.07,$ $\epsilon_{1xy \mathrm{iso}}=-1.33.$

$\frac{1}{a^{2}}\epsilon_{x}+ \frac{1}{c^{2}}\epsilon_{z}= \epsilon_{1,xz \text{iso}}\left( \frac{1}{a^{2}}+\frac{1}{c^{2}} \right)$,

$$\frac{1}{b^{2}}\epsilon_{y}+ \frac{1}{c^{2}}\epsilon_{z}= \epsilon_{1,yz \text{iso}}\left( \frac{1}{b^{2}}+\frac{1}{c^{2}} \right),$$

$$\frac{1}{a^{2}}\epsilon_{x}+ \frac{1}{b^{2}}\epsilon_{y}= \epsilon_{1,xy \text{iso}}\left( \frac{1}{a^{2}}+\frac{1}{b^{2}} \right).$$

In conclusion, we presented an approach to derive the high-order modes and eigen-permittivity relations of spherical and ellipsoid particles in the quasistatic regime. While isotropic structures have discrete eigenpermittivities, we showed that biaxial spheres and ellipsoids exhibit eigenpermittivity sum rules that relate between the permittivities of the different axes. Our results may apply to other fields of physics, such as thermal conduction and quasi-magnetostatics.

We assume that similarly to the isotropic inclusion case^25,26^ and the cylinder model in Ref. ^7^, high-order modes’ resonance condition appears in the scattering coefficient’s denominator. In Fig. S4 we plot $1/|\epsilon_{1}(\omega)-\epsilon_{1l}|$ and $1/|\epsilon_{1i}(\omega)/a_{i}^{2}+\epsilon_{1j}(\omega)/a_{j}^{2}-(\epsilon_{1ij \text{iso}}\left( \frac{1}{a_{i}^{2}}+\frac{1}{a_{j}^{2}} \right))|$ for the first ellipsoid when both resonances are on the long axes (A), when [100] is on a long axis and [010] is on the short axis (B), and when [100] and [010] are on the short and long axes respectively (C). The second option best agrees with the experimental results, where the strongest first and second-mode responses are approximately at the same frequency.


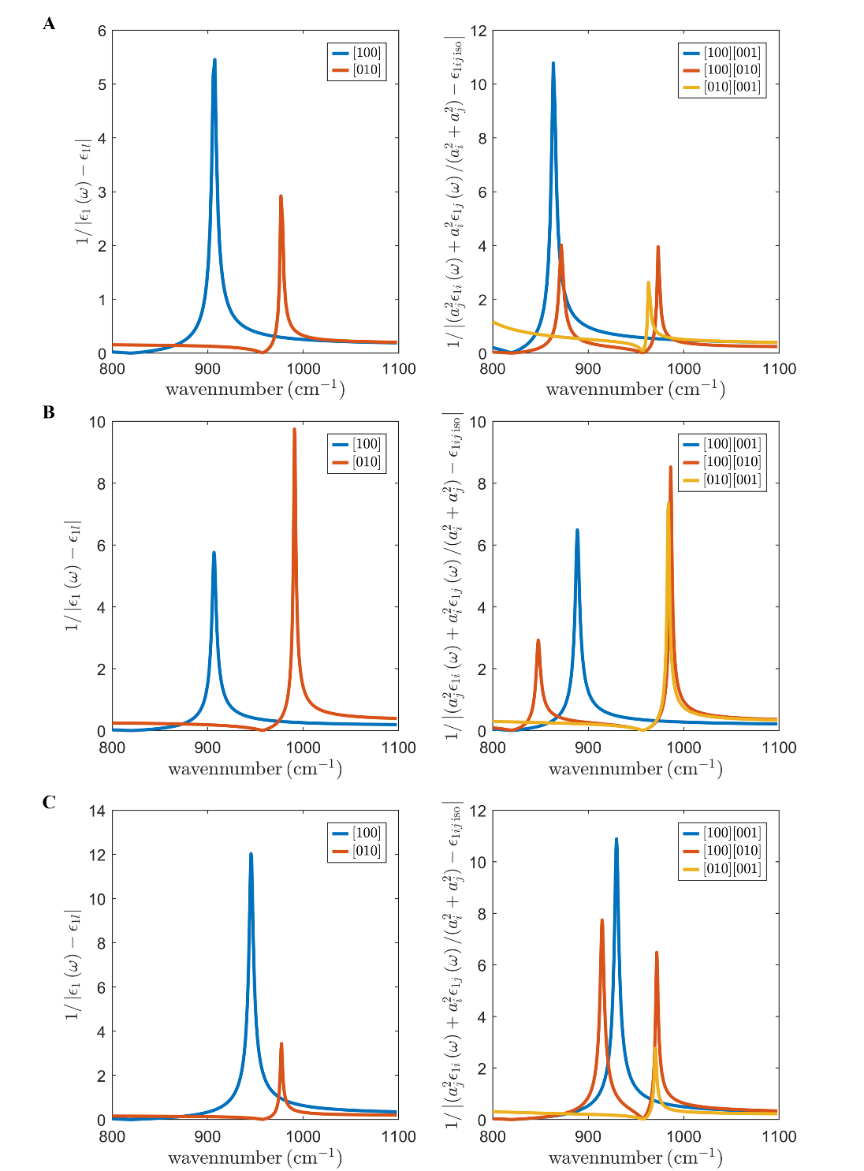


**Fig. S4. Three options for the spectrum for each crystal orientation with respect to the ellipsoid orientation for the oblate ellipsoid.** (**A**) Scattering amplitudes when both resonances are on the long axes. (**B**) Scattering amplitudes when [100] is on a long axis and [010] is on the short axis. (**C**) Scattering amplitudes when [100] is on the short axis and [010] on the long axis.

In Fig. S5 we plot $1/|\epsilon_{1}(\omega)-\epsilon_{1l}|$and $1/\left| \epsilon_{1i}(\omega)/a_{i}^{2}+\epsilon_{1j}(\omega)/a_{j}^{2}-\left( \epsilon_{1ij \text{iso}}\left( \frac{1}{a_{i}^{2}}+\frac{1}{a_{j}^{2}} \right) \right) \right|$ for the prolate ellipsoid when both resonances are on the short axes (A), when [100] is on a long axis and [010] is on the short axis (B), and when [100] is on the short axis and [010] is on a long axis (C). Here, the first option agrees with the experimental results. We also plotted the field distributions for both options, confirming this.


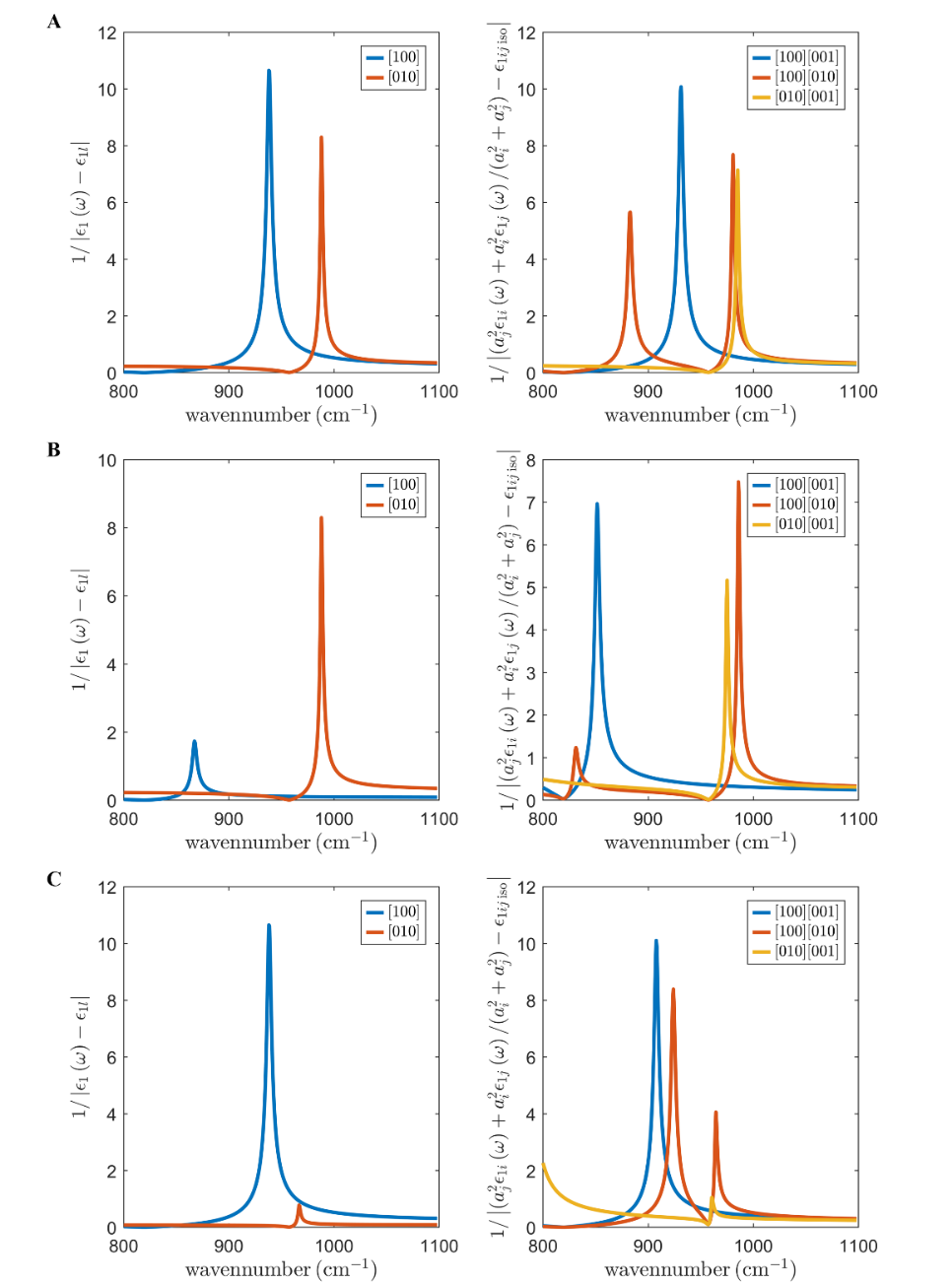


**Fig. S5. Three options for the spectrum for each crystal orientation with respect to the ellipsoid orientation for the second ellipsoid.** (**A**) Scattering amplitude when both resonances are on the short axes. (**B**) Scattering amplitudes when [100] is on a long axis and [010] is on the short axis. (**C**) Scattering amplitudes when [100] is on the short axis and [010] is on the long axis.

**Derivation of the anisotropic dipole mode of an anisotropic sphere**

We notice that an isotropic $l=1$ mode is still valid in this case, similarly to the scattering analysis in Ref. ^27^

$\psi_{l=1,m=0,r<a}\propto r\text{cos}\theta=z,$ where $\psi_{r>a}\propto\frac{1}{r^{2}}\text{cos}\theta,$ $\boldsymbol{E}_{l=1,m=0,r<a}\propto1\hat{z}.$ (S2.13)

We check the boundary conditions. The potential is continuous since it is a spherical harmonic, namely $\text{cos}\theta=\text{cos}\theta.$ We now impose continuous $D_{\perp}.$ Inside the sphere we project $E_{z}$ into the $r$ axis. Outside the sphere, we differentiate the field with respect to $r$ and multiply by $\epsilon_{2}$ and obtain

$-2\frac{1}{r^{3}}\text{cos}\theta\epsilon_{2}.$ (S2.14)

Thus, we get:

$\epsilon_{1z}\text{cos}\theta=-2\epsilon_{2}\text{cos}\theta,$ (S2.15)

$$\epsilon_{1z}=-2\epsilon_{2},$$

We get a similar relation to the isotropic case only in that the modes are in each axis according to the resonance there. Laplace’s equation in cartesian coordinates inside the sphere is satisfied for any $l=1$ mode since the field is constant.

Calculating $E_{z}$ for the near-field measurements

Sphere

The AFM tip scattered the electric field along its axis. We therefore calculate $E_{z}$ on the tip for the near and far field measurements.

Dipole mode

We assume that the excited-mode field is on the $z$axis of the crystal due to a resonance. This axis in principle can be different from the tip axis and we first assume that both are on the $z$axis. We calculate $E_{z}$of the mode, to account for the tip measurement:

$$\psi_{r>a}\propto\frac{1}{r^{2}}\text{cos}\theta,E_{z,r>a}\propto-\boldsymbol{e}_{r}\text{cos}\theta2r^{-3}-\boldsymbol{e}_{\theta}\frac{1}{r^{3}}\text{sin}\theta,$$

$E_{l=1,z}\propto-\frac{2}{r^{3}}\text{cos}^{2}\theta+\frac{1}{r^{3}}\text{sin}^{2}\theta$ (S2.16)

$$=\frac{1}{r^{3}}(-3\text{cos}^{2}\theta+\text{cos}^{2}\theta+\text{sin}^{2}\theta)=\frac{1}{r^{3}}(-3\text{cos}^{2}\theta+1)$$

$$=\frac{1}{r^{3}}(-3\text{cos}^{2}\theta+1.5-0.5)$$

$$=\frac{1}{r^{3}}(-\text{cos}2\theta-0.5),$$

where we used

$2\text{cos}^{2}\theta-1=\text{cos}2\theta.$ (S2.17)

For the near field, the mode amplitude is also proportional to $E_{z}$ ^4,26^ since the tip excites the mode. We thus get for the measurement:

$E_{tip near}=E_{\mathrm{mode}z}^{2}=\frac{1}{r^{6}}{(-3\text{cos}^{2}\theta+1)}^{2}$ (S2.18)

For the far-field measurement, the excitation strength does not depend on the tip location, and we get:

$E_{\mathrm{tip} \mathrm{far}}\propto\frac{1}{r^{3}}(-3\text{cos}^{2}\theta+1).$ (S2.19)

It is also multiplied by the polarization of the excitation:

$E_{\mathrm{tipfar}}=E_{\mathrm{inc}}\cdot E_{\mathrm{mode} \mathrm{inside}}.$ (S2.20)

We now verify the result:

$\psi_{r>a}=\frac{a^{2}}{r^{3}}z,E_{z}=\frac{a^{2}}{r^{3}}-3z\frac{a^{2}}{r^{4}}\frac{\partial r}{\partial z}=\frac{a^{2}}{r^{3}}-3z\frac{a^{2}}{r^{4}}\frac{\partial{(x^{2}+y^{2}+z^{2})}^{1/2}}{\partial z}=$ (S2.21)

$$\frac{a^{2}}{r^{3}}-3z\frac{a^{2}}{r^{4}}\frac{1}{2}\frac{2z}{r}=\frac{a^{2}}{r^{3}}-3\frac{a^{2}}{r^{3}}\frac{z^{2}}{r^{2}}=\frac{a^{2}}{r^{3}}-3\frac{a^{2}}{r^{3}}\text{cos}\theta^{2}\propto1-3z^{2}/a^{2}\propto1-3(a^{2}-x^{2}-y^{2})/a^{2},$$

and we have an agreement.

Second-order mode

We calculate $E_{r>a}$ from the boundary conditions (up to normalization factors):

$$\psi=xz,$$

$$\boldsymbol{E}_{\mathrm{ins}}=z\boldsymbol{e}_{x}+x\boldsymbol{e}_{z},$$

$$\boldsymbol{D}_{\mathrm{ins}}=\epsilon_{x}z\boldsymbol{e}_{x}+\epsilon_{z}x\boldsymbol{e}_{z},$$

$$\boldsymbol{D}_{\mathrm{in}}\cdot\boldsymbol{e}_{r}=\frac{1}{a}(x\epsilon_{x}z+z\epsilon_{z}x)\Rightarrow E_{r\mathrm{out}}=\boldsymbol{D}_{\mathrm{in}}\cdot\boldsymbol{e}_{r}=\frac{1}{a}xz(\epsilon_{x}+\epsilon_{z}),$$

$$E_{r \mathrm{ins}}=\frac{2}{a}xz,$$

$E_{\parallel\mathrm{out}}=E_{\parallel\mathrm{ins}}=\boldsymbol{E}_{\mathrm{ins}}-E_{\perp\mathrm{ins}}=z\boldsymbol{e}_{x}+x\boldsymbol{e}_{z}-\frac{2}{a}(xz)\boldsymbol{e}_{r}.$ (S2.22)

$$E_{\perp\mathrm{out}}=-\frac{3}{a}(xz)\boldsymbol{e}_{r}.$$

$$E_{\mathrm{out}}=z\boldsymbol{e}_{x}+x\boldsymbol{e}_{z}-\frac{5}{a}(xz)\frac{1}{a}(x\boldsymbol{e}_{x}+y\boldsymbol{e}_{y}+z\boldsymbol{e}_{z}),$$

$$E_{z}=x\left( 1-\frac{5}{a^{2}}z^{2} \right),E_{z,a=1}=x(1-5(1-x^{2}-y^{2})),$$

$$E_{z,a=2}=x(1-5/4(1-x^{2}-y^{2})).$$

Alternatively, we can calculate $E_{z}$ from the expression for $r>a$:

$\frac{\psi_{2}^{1}-\psi_{2}^{-1}}{2}=\frac{a^{3}}{r^{3}}\text{cos}\theta\text{sin}\theta\text{cos}\phi$ (S2.23)

$$E_{z}=\frac{\partial}{\partial z}\frac{a^{3}}{r^{5}}xz=-5\frac{a^{3}}{r^{6}}\frac{x}{r}xz+\frac{a^{3}}{r^{5}}x\overset{r=a}{\to}-5\frac{x^{2}z}{a^{4}}+\frac{1}{a^{2}}x$$

where $\frac{\partial r}{\partial x}=\frac{x}{r},$ and we get agreement.

The predicted measurement for a general tip orientation

The easiest way to project the field into the tip axis would be to take the inner product:

$E_{\mathrm{tip}}=\boldsymbol{E}_{\mathrm{mode}}\cdot\boldsymbol{e}_{\mathrm{tip}}=(E_{x},E_{y},E_{z})\cdot(\text{sin}\theta\text{cos}\phi,\text{sin}\theta\text{sin}\phi,\text{cos}\theta).$ (S2.24)

Anisotropic ellipsoid

We proceed in the first approach of calculating the field using the boundary conditions. We project $\boldsymbol{D}$ inside the ellipsoid on the vector perpendicular to the ellipsoid interface to calculate the mode outside the ellipsoid. For simplicity, we start with an ellipse:

$$x=a\text{cos}\theta,y=b\text{sin}\theta,$$

$\frac{dy}{dx}=\frac{\frac{dy}{dt}}{\frac{dx}{dt}}=\frac{b\text{cos}\theta}{-a\text{sin}\theta}\Rightarrow m_{\parallel}=\left( -\frac{a}{\text{cos}\theta},\frac{b}{\text{sin}\theta} \right),m_{\perp}=\frac{a}{b}\text{tan}\theta,$ (S2.25)

$$v_{\perp}\propto\left( \frac{\text{cos}\theta}{a},\frac{\text{sin}\theta}{b} \right)=\left( \frac{x}{a^{2}},\frac{y}{b^{2}} \right),$$

where $v_{\perp}$ is the perpendicular vector and $m_{\parallel,}m_{\perp}$are the slopes parallel and perpendicular to the ellipse.

Dipole mode

Generalizing to an ellipsoid, we have $v_{\perp}=\frac{\left( \frac{x}{a^{2}},\frac{y}{b^{2}},\frac{z}{c^{2}} \right)}{|n|},|n|=\sqrt{\left( \frac{x}{a^{2}} \right)^{2}+\left( \frac{y}{b^{2}} \right)^{2},\left( \frac{z}{c^{2}} \right)^{2}}$ and get for the dipole mode:

$$\boldsymbol{D}_{\perp}=E_{1}\epsilon_{1z}\left[ \boldsymbol{e}_{z}\cdot\left( \frac{x}{a^{2}},\frac{y}{b^{2}},\frac{z}{c^{2}} \right) \right]\left( \frac{x}{a^{2}},\frac{y}{b^{2}},\frac{z}{c^{2}} \right)/\left| n \right|^{2}=E_{1}\epsilon_{1z}\frac{z}{c^{2}}\left( \frac{x}{a^{2}},\frac{y}{b^{2}},\frac{z}{c^{2}} \right)/\left| n \right|^{2}$$

$E_{\perp\mathrm{outside}}\cdot\boldsymbol{e}_{z}=E_{1}\frac{\epsilon_{1z}}{\epsilon_{2}}\frac{z^{2}}{c^{4}}/\left| n \right|^{2},$ (S2.26)

$$E_{\perp\mathrm{outside}}=E_{1}\frac{\epsilon_{1z}}{\epsilon_{2}}\frac{z}{c^{2}}\left( \frac{x}{a^{2}},\frac{y}{b^{2}},\frac{z}{c^{2}} \right)/\left| n \right|^{2}.$$

We subtract the perpendicular field from the total field inside (and outside) the ellipsoid to get the parallel field and deduce the total field outside:

$$E_{\parallel}=E_{1}\left[ \boldsymbol{e}_{z}-\frac{z}{c^{2}}\left( \frac{x}{a^{2}},\frac{y}{b^{2}},\frac{z}{c^{2}} \right)/\left| n \right|^{2} \right],$$

$E_{total outside}=E_{1}\left[ \boldsymbol{e}_{z}+\left( \frac{\epsilon_{1z}}{\epsilon_{2}}-1 \right)\frac{z}{c^{2}}\left( \frac{x}{a^{2}},\frac{y}{b^{2}},\frac{z}{c^{2}} \right)/\left| n \right|^{2} \right],$ (S2.27)

$$E_{z\mathrm{total}}\propto1+\frac{\left( -1+\epsilon_{1z} \right)z^{2}}{c^{4}}/\left| n \right|^{2}.$$

To compare this expression to the expression for a sphere, we substitute $\epsilon_{1z}=-2 \mathrm{and}$ get:

$E_{z}\propto1-3z^{2}/r^{2}=1-3\text{cos}^{2}\theta,$ (S2.28)

which is equal for $r=1$ to the sphere expression $\frac{1}{r^{3}}(-3\text{cos}^{2}\theta+1).$ We write the measured field for a general tip orientation:

$$E_{\mathrm{tip}}=\left[ \left( 0,0,1 \right)+\left( \frac{\epsilon_{1z}}{\epsilon_{2}}-1 \right)\frac{z}{c^{2}}\left( \frac{x}{a^{2}},\frac{y}{b^{2}},\frac{z}{c^{2}} \right) \right]/\left| n \right|^{2}\cdot(\text{sin}\theta\text{cos}\phi,\text{sin}\theta\text{sin}\phi,\text{cos}\theta),$$

$E_{\mathrm{tip}}=\left[ \text{cos}\theta+\left( \frac{\epsilon_{1z}}{\epsilon_{2}}-1 \right)\frac{z}{c^{2}}\left( \frac{x}{a^{2}}\text{sin}\theta\text{cos}\phi+\frac{y}{b^{2}}\text{sin}\theta\text{sin}\phi+\frac{z}{c^{2}}\text{cos}\theta\right) \right]^{2}.$ (S2.29)

We compare the result with $\theta=0$ to the previous

$E_{\mathrm{tip}}=\left[ 1+\left( \frac{\epsilon_{1z}}{\epsilon_{2}}-1 \right)\frac{z}{c^{2}}\left( \frac{z}{c^{2}} \right) \right]^{2}.$ (S2.30)

For $\theta=\pi/4$ we get:

$E_{\mathrm{tip}}=\left[ \frac{1}{\sqrt{2}}+\left( \frac{\epsilon_{1z}}{\epsilon_{2}}-1 \right)\frac{z}{c^{2}}\left( \frac{1}{\sqrt{2}}\frac{x}{a^{2}}+\frac{1}{\sqrt{2}}\frac{z}{c^{2}} \right) \right].$ (S2.31)

Finally, we calculate $E_{z}$ for a dipole resonance on $y$ axis:

$$D_{\perp}=E_{1}\epsilon_{1y}\left[ \boldsymbol{e}_{y}\cdot\left( \frac{x}{a^{2}},\frac{y}{b^{2}},\frac{z}{c^{2}} \right) \right]\left( \frac{x}{a^{2}},\frac{y}{b^{2}},\frac{z}{c^{2}} \right)/\left| n \right|^{2}=\epsilon_{1y}\frac{y}{b^{2}}\left( \frac{x}{a^{2}},\frac{y}{b^{2}},\frac{z}{c^{2}} \right)/\left| n \right|^{2},$$

$E_{\perp\mathrm{outside}}=\frac{\epsilon_{1y}}{\epsilon_{2}}\frac{y}{b^{2}}\left( \frac{x}{a^{2}},\frac{y}{b^{2}},\frac{z}{c^{2}} \right)/\left| n \right|^{2},$ (S2.32)

$$E_{\parallel}=E_{1}[\boldsymbol{e}_{y}-\frac{y}{b^{2}}\left( \frac{x}{a^{2}},\frac{y}{b^{2}},\frac{z}{c^{2}} \right)/\left| n \right|^{2}],$$

$$E_{z \mathrm{outside}}=\frac{yz}{b^{2}c^{2}}\left( \frac{\epsilon_{1y}}{\epsilon_{2}}-1 \right)/\left| n \right|^{2}.$$

Second-order mode

We now perform the calculation for the second-order mode of an ellipsoid:

$$\psi_{\text{ins}}=xz,$$

$\boldsymbol{E}_{\mathrm{ins}}=z\boldsymbol{e}_{x}+x\boldsymbol{e}_{z},$

$$\boldsymbol{D}_{\mathrm{ins}}=\epsilon_{x}z\boldsymbol{e}_{x}+\epsilon_{z}x\boldsymbol{e}_{z},$$

$$\boldsymbol{D}_{\mathrm{in}}\cdot\boldsymbol{e}_{\perp}=\left( \frac{1}{a^{2}}x\epsilon_{x}z+\frac{1}{c^{2}}z\epsilon_{z}x \right)/\left| n \right|=E_{\perp\mathrm{out}},$$

$$E_{\perp\mathrm{ins}}=\left( \frac{1}{a^{2}}+\frac{1}{c^{2}} \right)xz/\left| n \right|,$$

$$\boldsymbol{E}_{\parallel\mathrm{out}}=\boldsymbol{E}_{\parallel\mathrm{ins}}=\boldsymbol{E}_{\mathrm{ins}}-E_{\perp\mathrm{ins}}=z\boldsymbol{e}_{x}+x\boldsymbol{e}_{z}-\left( \frac{1}{a^{2}}+\frac{1}{c^{2}} \right)xz\boldsymbol{e}_{\perp}/\left| n \right|,$$

$\boldsymbol{E}_{\mathrm{out}}=z\boldsymbol{e}_{x}+x\boldsymbol{e}_{z}+xz\left( \frac{1}{a^{2}}\epsilon_{x}+\frac{1}{c^{2}}\epsilon_{z}-\left( \frac{1}{a^{2}}+\frac{1}{c^{2}} \right) \right)\left( \frac{1}{a^{2}}x\boldsymbol{e}_{x}+\frac{1}{b^{2}}y\boldsymbol{e}_{y}+\frac{1}{c^{2}}z\boldsymbol{e}_{z} \right)/\left| n \right|^{2},$ (S2.33)

$$E_{\mathrm{tip}}=\left[ z\text{sin}\theta\text{cos}\phi+x\text{cos}\theta+xz\left( \frac{1}{a^{2}}\epsilon_{x}+\frac{1}{c^{2}}\epsilon_{z}-\left( \frac{1}{a^{2}}+\frac{1}{c^{2}} \right) \right)\left( \frac{1}{a^{2}}x\text{sin}\theta\text{cos}\phi+\frac{1}{b^{2}}y\text{sin}\theta\text{sin}\phi+\frac{1}{c^{2}}z\text{cos}\theta\right) \right]^{2}$$

$$\boldsymbol{E}_{\mathrm{out}}=z\boldsymbol{e}_{x}+x\boldsymbol{e}_{z}+\frac{xz}{d}\left( \frac{1}{a^{2}}x\boldsymbol{e}_{x}+\frac{1}{b^{2}}y\boldsymbol{e}_{y}+\frac{1}{c^{2}}z\boldsymbol{e}_{z} \right)/\left| n \right|^{2},$$

$$\frac{1}{d}=\frac{1}{a^{2}}(\epsilon_{x}-1)+\frac{1}{c^{2}}(\epsilon_{z}-1),$$

$$\boldsymbol{E}_{\mathrm{out}}=z\left( 1+\frac{1}{a^{2}d\left| n \right|^{2}}x^{2} \right)\boldsymbol{e}_{x}+\frac{1}{b^{2}d\left| n \right|^{2}}xyz\boldsymbol{e}_{y}+x\left( 1+\frac{1}{c^{2}d\left| n \right|^{2}}z^{2} \right)\boldsymbol{e}_{z},$$

$$E_{\mathrm{out}}^{2}=\left[ z\left( 1+\frac{1}{a^{2}d\left| n \right|^{2}}x^{2} \right) \right]^{2}+\left[ \frac{1}{b^{2}d\left| n \right|^{2}}xyz \right]^{2}+\left[ x\left( 1+\frac{1}{c^{2}d\left| n \right|^{2}}z^{2} \right) \right]^{2}.$$

compared to the sphere we get

$\frac{1}{d}=\frac{1}{a^{2}}(\epsilon_{x}-1)+\frac{1}{c^{2}}(\epsilon_{z}-1)=-5/a^{2},$ (S2.34)

$$\boldsymbol{E}_{\mathrm{out}}=z\boldsymbol{e}_{x}+x\boldsymbol{e}_{z}-\frac{5}{a^{2}}(xz)\frac{1}{a^{2}}(x\boldsymbol{e}_{x}+y\boldsymbol{e}_{y}+z\boldsymbol{e}_{z}),$$

and we get a good agreement.

Similarly,

$$\psi=yz,$$

$$\frac{1}{d_{yz}}=\frac{1}{b^{2}}(\epsilon_{y}-1)+\frac{1}{c^{2}}(\epsilon_{z}-1)$$

$\boldsymbol{E}_{\mathrm{out}}=z\boldsymbol{e}_{y}+y\boldsymbol{e}_{z}+\frac{yz}{d_{yz}}\left( \frac{1}{a^{2}}x\boldsymbol{e}_{x}+\frac{1}{b^{2}}y\boldsymbol{e}_{y}+\frac{1}{c^{2}}z\boldsymbol{e}_{z} \right)/\left| n \right|^{2},$ (S2.35)

$$\psi=xy,$$

$$\frac{1}{d_{xy}}=\frac{1}{b^{2}}(\epsilon_{y}-1)+\frac{1}{a^{2}}(\epsilon_{x}-1)$$

$$\boldsymbol{E}_{\mathrm{out}}=x\boldsymbol{e}_{y}+y\boldsymbol{e}_{x}+\frac{xy}{d_{xy}}\left( \frac{1}{a^{2}}x\boldsymbol{e}_{x}+\frac{1}{b^{2}}y\boldsymbol{e}_{y}+\frac{1}{c^{2}}z\boldsymbol{e}_{z} \right)/\left| n \right|^{2}.$$

To verify the theory, we performed COMSOL simulations of the oblate ellipsoid with the same semiaxis ratio as in the experiment and smaller by a factor of 5, excited by an oscillating dipole. The simulations were performed using a frequency sweep in the range 900-1000 1/cm, similarly to the experiment. In Figure S6 we present the resonances and the corresponding field distributions obtained in the simulation and compare them to the analytic results with very good agreement.


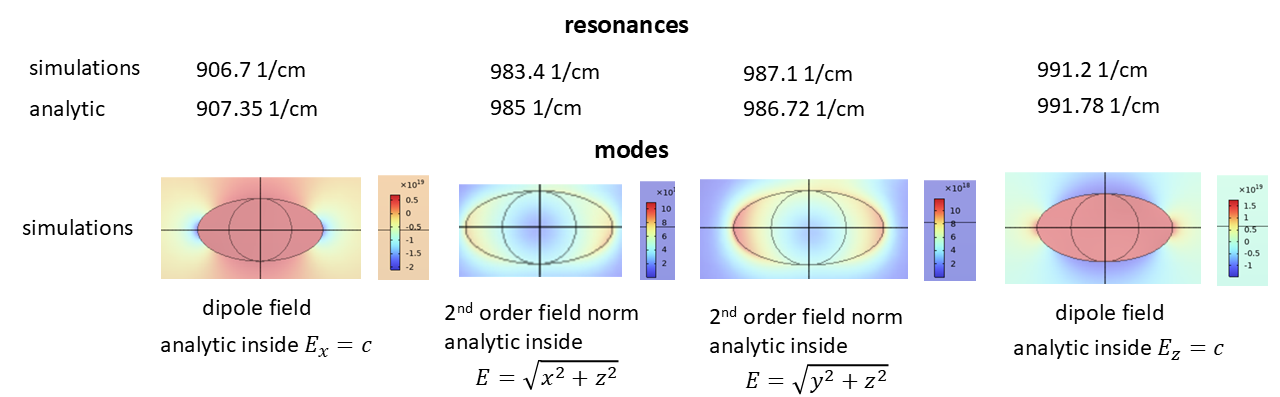


***Fig. S6*** ***Comparison of resonances and modes obtained analytically to the ones calculated in COMSOL simulations for the oblate ellipsoid****. The simulated oblate ellipsoid had the same semiaxis ratios and was smaller by a factor of 5 compared to the experiment. The simulations were performed for two dipole locations in the vicinity of the nanoellispoid with the same resonances and field distributions. The field magnitude is presented on the xy and xz planes.*

**S3 - nano-ellipsoid sample**

One of the primary samples presented in this study is an α-MoO_3_ nanoellipsoid. 7A shows a SEM micrograph of the sample with a measured diameter of ~210 nm. Fig. S7B shows an energy-dispersive X-ray spectrum (EDXS) taken from the sample area, with peaks for Mo and O (Si, C, and Al are substrate signals). In Fig. S7C, we present an AFM topological scan of the sample with a similar shape to the SEM. In Fig. S7D we plot the averaged radial height profile from the center of the sample. The height profile has a maximum height of ~115 nm. The combined SEM and AFM measurements give a nano-ellipsoid diameter dimensions of 210/115 nm and an oblate shape.


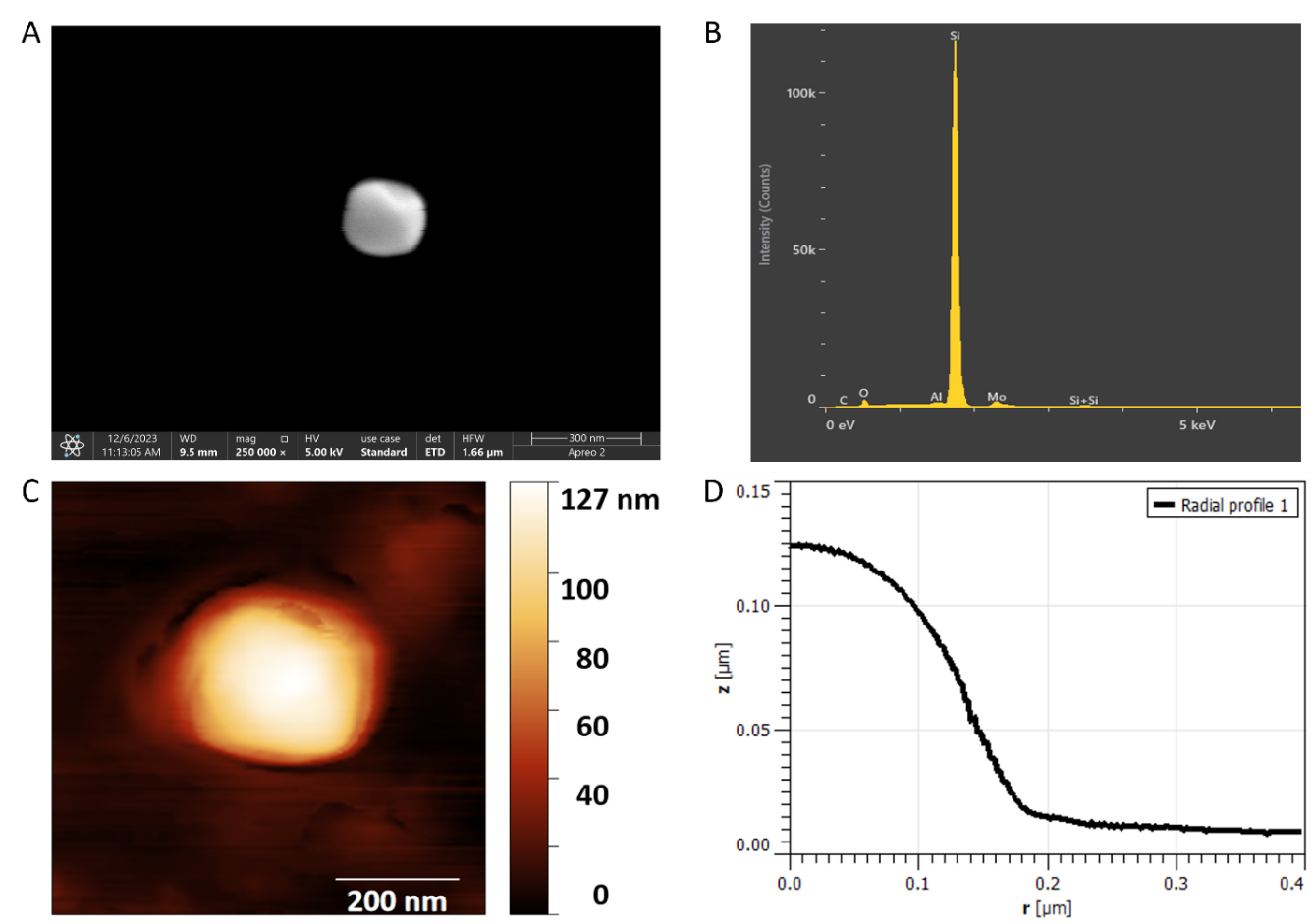


**Fig. S*7*. SEM and AFM analysis of the nano-ellipsoid sample.** (**A**) Environmental scanning electron microscope micrograph of the nanoellipsoid. The measured diameter of the ellipsoid was 210 nm. (**B**) The energy-dispersive X-ray spectrum of the nano-ellipsoid particle shows Mo and O. The carbon, silicon, and aluminium signals are from the substrate. (**C**) AFM topology z scan of the nano-ellipsoid sample. (**D**) Averaged radial AFM height profile of the nano-ellipsoid sample showing a maximum height of ~115 nm.

The near-field amplitude and phase spectra of the α-MoO_3_ nanoellipsoid were measured and averaged on the area of the particle using the AFM topological measurement to detect the edges, as shown in Fig. S8A-B. The amplitude spectra show a double step, indicating another possible resonance at about ~980 cm^-1^.


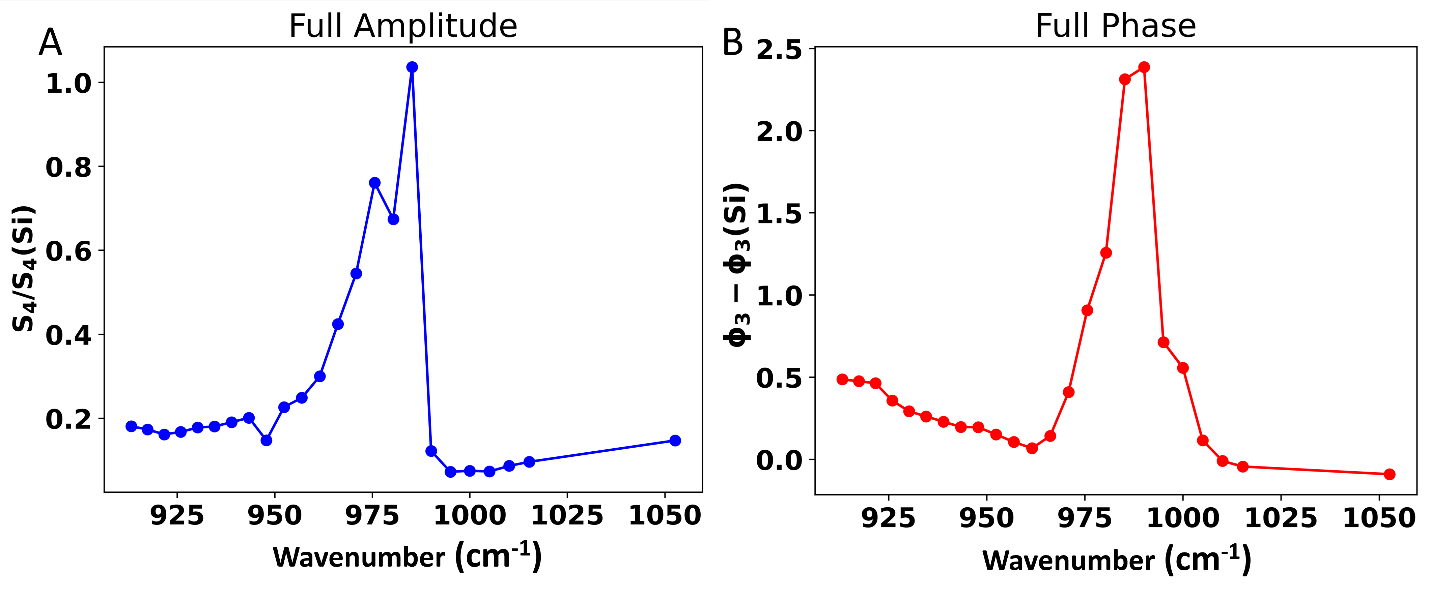


**Fig. S8. Near-field amplitude and phase spectra of the α-MoO3 prolate nanollipsoid from Fig. S6 measured via s-SNOM across nano ellipsoid area as determined by AFM topology measurements.** (**A**) 3rd harmonic near-field amplitude normalized with respect to the Si substrate. (**B**) 3rd harmonic near-field phase normalized to the Si substrate signal.

**S4 - Additional nano-ellipsoid sample**

A second α-MoO_3_ nanoellipsoid sample was measured. Figure S9A shows the AFM topology measurement of the sample with a height of ~78 and a similar radial profile (Fig. S9B) to the sample in Fig**.** S7D, which indicates dimensions of 220/78 nm. Figures S9C-D shows the s-SNOM spectrum phase and amplitude, respectively. The s-SNOM phase point spectroscopy spectrum has a single sharp peak at 990 cm^-1^. This peak is sharper than the other samples, with a calculated Q-factor of ~285. The s-SNOM amplitude spectrum shows two resonances, one at ~975 cm^-1^ and another at ~990 cm^-1^, which matches the resonance peaks observed in the phase spectra.


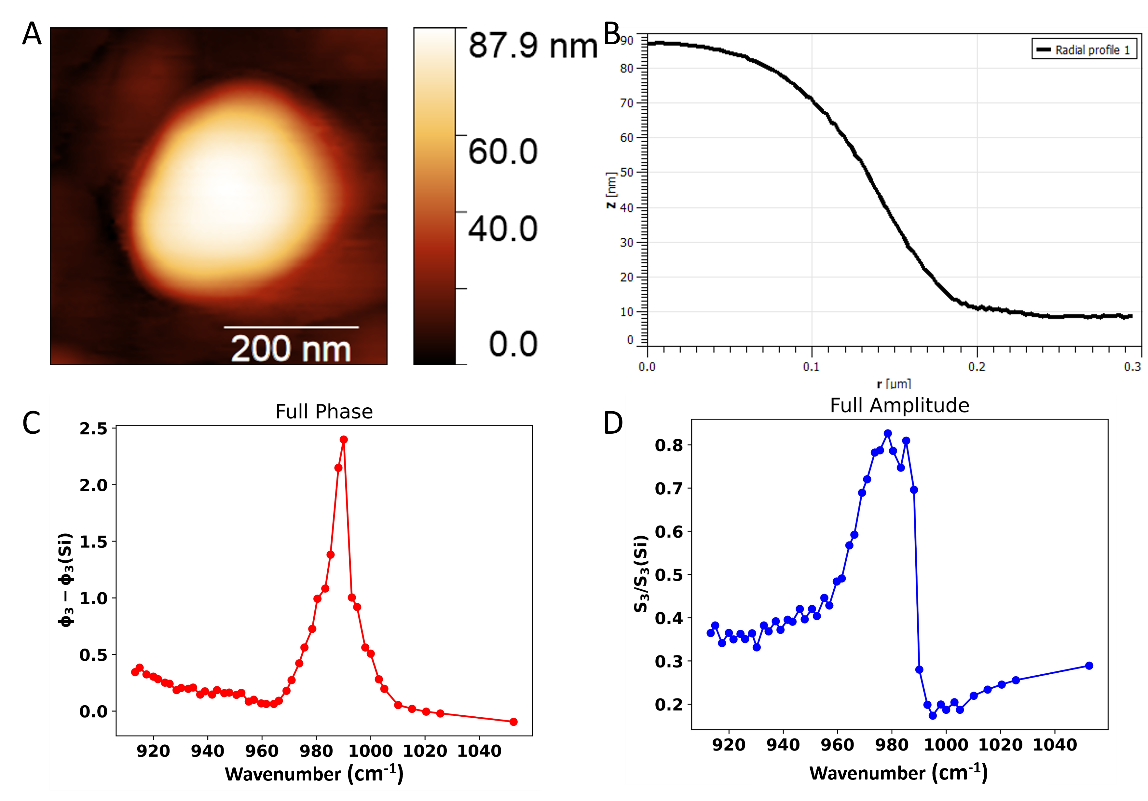


**Fig. S9.** **Data from an additional nanoellipsoid sample.** (**A**) AFM topology $z$ scan of the nanoellipsoid sample. (**B**) Averaged radial AFM height profile of the nanoellipsoid sample with a maximum height of ~78 nm and a similar profile to the sample shown in Fig. S4. (**C**) s-SNOM phase spectrum of the sample with a single resonance peak at 990 cm^-1^ and a Q factor of ~285. (**D)** s-SNOM amplitude spectrum of the sample with two close resonances at ~975 cm^-1^ and ~990 cm^-1^.

To test the validity of the method used in our work to separate the round-trip and direct contributions, we performed two s-SNOM hyperspectral measurements on the particle shown in Fig. S9. In the first scan, the in-plane polarization component of the incident beam was aligned along the [100] crystal axis, thereby directly exciting the [100]/[010] mode. In the second scan, the polarization was oriented perpendicular to this mode, along the [001] crystal direction. The round-trip component remains identical for both polarization directions, as it depends only on the out-of-plane polarization interacting with the AFM tip. However, the direct contribution shows a significant drop in magnitude when the polarization is no longer aligned to excite the [100]/[010] mode directly. This demonstrates that we can successfully separate the in-plane and out-of-plane modes of the particle, even when they spectrally overlap.

**
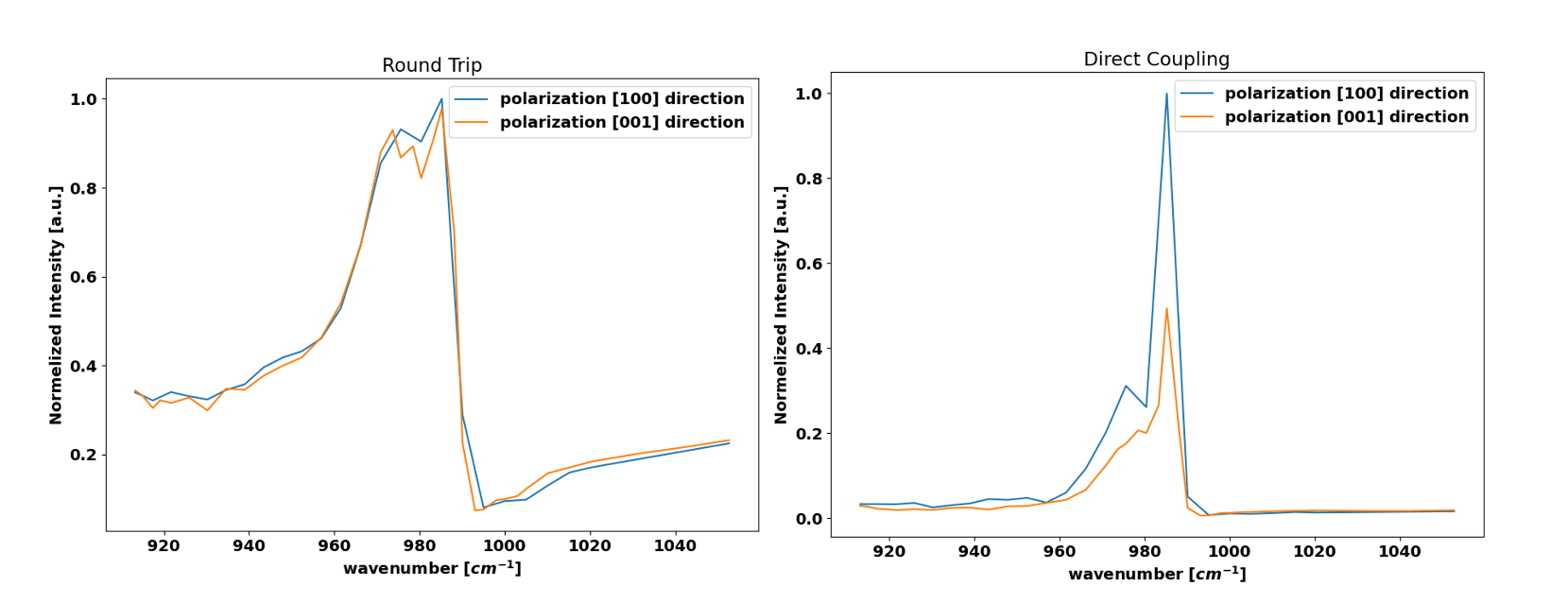
**

**Fig. S10. Symmetric and antisymmetric near-field spectrum of additional oblate nanoellipsoid sample from Fig S9.** The measurements were performed with the far-field in-plane polarization aligned with the sample [100] crystal axis, and the other with the far-field in-plane polarization perpendicular to the [001] crystal direction. As can be seen, the round-trip components are independent of the in-plane polarization as they are either directly excited from the tip (round trip) or as a result of the out-of-plane-oriented [010] dipole mode. The direct coupling components for the [001] polarization measurement has a ~0.5 in magnitude drop as compared to the [100] polarization measurement, as the change in polarization greatly reduces excitation of the [100][001] mode by the far field.

**S5: prolate ellipsoid sample**

The α-MoO_3_ prolate ellipsoid sample presented in this work was measured using SEM and AFM. Fig. **S**11A and B show the AFM topology measurement results of the sample with a height of ~120 nm and a width cross-section similar to the measured nanoellipsoids. Fig. **S**11C-D show the SEM results for the sample. Fig. **S**11C shows the SEM micrograph length/width dimensions of ~380/120. Fig. **S**11D displays the EDXS spectrum from the sample area with peaks for Mo and O (Si, C, and Al are substrate signals).

The far-field absorption spectrum of the (200,60,60) nm prolate ellipsoid was simulated using FDTD. In the simulation, the prolate ellipsoid is illuminated via a plane wave traveling in the [001] crystal directions of the particle with polarization in the prolate ellipsoid small cross-section plane aligned at 45° with respect to both the [100] and [010] crystal directions. As a result, both phonon dipole peaks are visible in the spectrum, see Fig. S11.


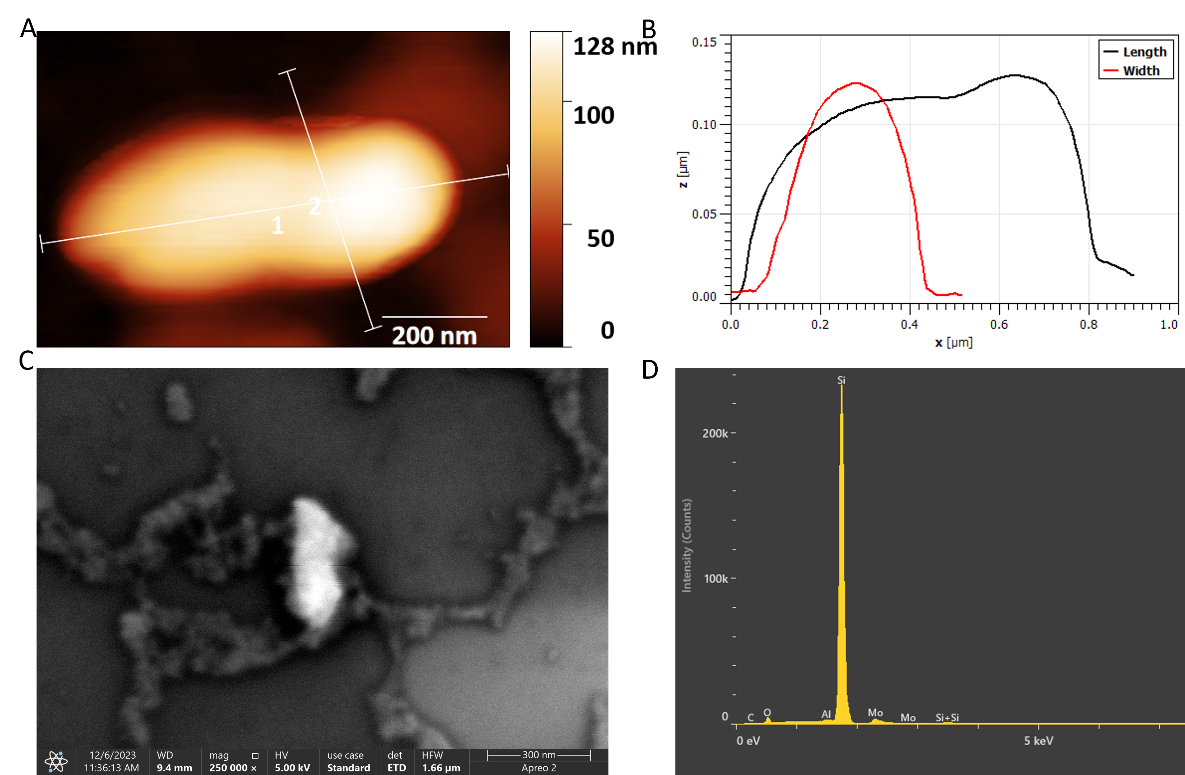


**Fig. S11. Data from the prolate ellipsoid sample.** (**A**) AFM topology z scan of the prolate ellipsoid sample. (**B**) Length and width profile on nano-bar (the profile lines are marked on (a) as 1 – length and 2 – width). (**C**) SEM micrograph of the prolate ellipsoid. The measured diameter of the ellipsoid was 210 nm. (**D**) The energy-dispersive X-ray spectrum of the prolate ellipsoid particle, showing Mo and O. Carbon, silicon, and aluminum are from the substrate.

**
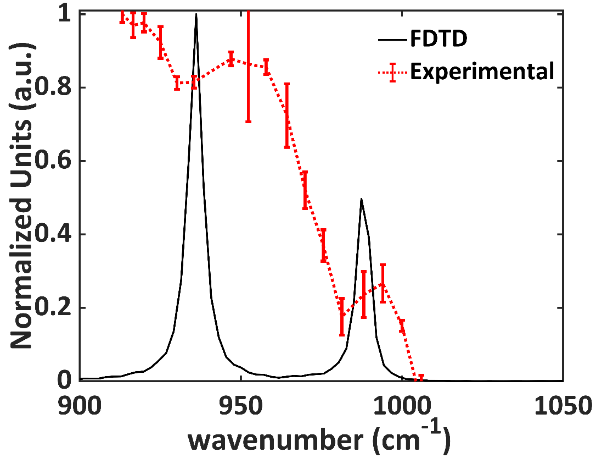
**

**Fig. S12.** Far-field FDTD simulation of α-MoO_3_ prolate ellipsoid (200,60,60) nm absorption spectrum compared to the normalized near-field phase spectrum measured using s-SNOM at the ellipsoid center.

References

1. Beitner, D. *et al.* Mid-Infrared Mapping of Four-Layer Graphene Polytypes Using Near-Field Microscopy. *Nano Lett* **09**, 28 (2023).

2. Beitner, D., Carmeli, I., Zalevsky, Z., Richter, S. & Suchowski, H. Coupled Molecular Emitters in Superstructures Interact with Plasmonic Nanoparticles. *Adv Photonics Res* **3**, 2100334 (2022).

3. Nečas, D. & Klapetek, P. Gwyddion: An open-source software for SPM data analysis. *Central European Journal of Physics* **10**, 181–188 (2012).

4. Tamagnone, M. *et al.* Ultra-confined mid-infrared resonant phonon polaritons in van der Waals nanostructures. *Sci Adv* **4**, (2018).

5. Agranovich, V. M. & Ginzburg, V. *Crystal Optics with Spatial Dispersion, and Excitons*. (Springer Berlin Heidelberg, Berlin, Heidelberg, 1984). doi:10.1007/978-3-662-02406-5.

6. Yariv, Amnon. & Yeh, P. *Optical Waves in Crystals : Propagation and Control of Laser Radiation*. (United States: N. p., 2003).

7. Farhi, A. & Dogariu, A. Coupling of electrodynamic fields to vibrational modes in helical structures. *Phys Rev A  (Coll Park)* **103**, 023523 (2021).

8. Zheng, Z. *et al.* A mid-infrared biaxial hyperbolic van der Waals crystal. *Sci Adv* **5**, (2019).

9. He, M. *et al.* Guided Polaritons along the Forbidden Direction in MoO3 with Geometrical Confinement. *Nano Lett* **23**, 5041 (2023).

10. Barcelos, I. D. *et al.* Ultrabroadband Nanocavity of Hyperbolic Phonon-Polaritons in 1D-Like α-MoO3. *ACS Photonics* **8**, 3017–3026 (2021).

11. Kumar, S. *et al.* Synthesis of α-MoO3 nanofibers for enhanced field-emission properties. *researchgate.netSKS Patel, K Dewangan, SK Srivastav, NK Verma, P Jena, AK Singh, NS GajbhiyeAdv. Mater. Lett., 2018•researchgate.net* **2018**, 585–589 (2018).

12. Wang, Z., Madhavi, S., Chemistry, X. L.-T. J. of P. & 2012, undefined. Ultralong α-MoO3 Nanobelts: Synthesis and Effect of Binder Choice on Their Lithium Storage Properties. *ACS PublicationsZ Wang, S Madhavi, XW LouThe Journal of Physical Chemistry C, 2012•ACS Publications* **116**, 12508–12513 (2012).

13. Nitharwal, R. K. *et al.* Manifestation of anharmonicities in terms of Fano scattering and phonon lifetime of scissors modes in α-MoO 3. *Physical Chemistry Chemical Physics* **26**, 17892–17901 (2024).

14. Vo Huu, T. *et al.* Nanorod structure tuning and defect engineering of MoO x for high-performance SERS substrates. *Nanoscale* **16**, 22297–22311 (2024).

15. Lupan, O. Investigation of optical properties and electronic transitions in bulk and nano-microribbons of molybdenum trioxide. *iopscience.iop.orgO Lupan, V Trofim, V Cretu, I Stamov, NN Syrbu, I Tiginyanu, YK Mishra, R AdelungJournal of Physics D: Applied Physics, 2014•iopscience.iop.org* (2014) doi:10.1088/0022-3727/47/8/085302.

16. Calvo-Mola, C., Torres-Costa, V., Compounds, Y. G.-… A. and & 2022, undefined. Temperature dependence of Raman and photoluminescence spectra of pure and high-quality MoO3 synthesized by hot wall horizontal thermal evaporation. *Elsevier, V Torres-Costa, Y González, A Ruediger, M Sánchez, G Santana, G Contreras-Puente…Journal of Alloys and Compounds, 2022•Elsevier*.

17. Dewangan, K. *et al.* Synthesis and characterization of single-crystalline α-MoO3 nanofibers for enhanced Li-ion intercalation applications. *pubs.rsc.orgK Dewangan, NN Sinha, PK Sharma, AC Pandey, N Munichandraiah, NS GajbhiyeCrystEngComm, 2011•pubs.rsc.org* doi:10.1039/c0ce00271b.

18. Kong, H. *et al.* Growth and physical properties of large MoO3 single crystals. *J Mater Sci* **51**, 8928–8934 (2016).

19. Nunna, G. P. *et al.* Biogenic Synthesis of High‐Performance α‐MoO3 Nanoparticles from Tryptophan Derivatives for Antimicrobial Agents and Electrode Materials of Supercapacitors. *Wiley Online LibraryGP Nunna, HK Siddarapu, VVJ Nimmagadda, MH Obili, TJ Ko, J Lim, J ChoiInternational Journal of Energy Research, 2023•Wiley Online Library* **2023**, (2023).

20. Pascariu, P., Homocianu, M., Olaru, N., Nanomaterials, A. A.- & 2020, undefined. New Electrospun ZnO:MoO3 Nanostructures: Preparation, Characterization and Photocatalytic Performance. *mdpi.comP Pascariu, M Homocianu, N Olaru, A Airinei, O IonescuNanomaterials, 2020•mdpi.com*.

21. Gowtham, B., Ponnuswamy, V., Pradeesh, G., Chandrasekaran, J. & Aradhana, D. MoO3 overview: hexagonal plate-like MoO3 nanoparticles prepared by precipitation method. *Journal of Materials Science: Materials in Electronics* **29**, 6835–6843 (2018).

22. Mestl, G., Ruiz, P., Delmon, B. & Knözinger, H. Oxygen-Exchange Properties of MoO3: An in situ Raman Spectroscopy Study. *J Phys Chem* **98**, 11269–11275 (1994).

23. Dieterle, M., Weinberg, G. & Mestl, G. Raman spectroscopy of molybdenum oxides. *Physical Chemistry Chemical Physics* **4**, 812–821 (2002).

24. Guzatov, D. V., Klimov, V. V. & Pikhota, M. Yu. Plasmon oscillations in ellipsoid nanoparticles: Beyond dipole approximation. *Laser Phys* **20**, 85–99 (2010).

25. Bergman, D. J. Dielectric constant of a two-component granular composite: A practical scheme for calculating the pole spectrum. *Phys Rev B* **19**, 2359 (1979).

26. Farhi, A. & Bergman, D. J. Eigenstate expansion of the quasistatic electric field of a point charge in a spherical inclusion structure. *Phys Rev A  (Coll Park)* **96**, 043806 (2017).

27. Bohren, C. F. & Huffman, D. R. *Absorption and Scattering of Light by Small Particles*. *Absorption and Scattering of Light by Small Particles* (Wiley, 1998). doi:10.1002/9783527618156.
